# RNA sequencing indicates widespread conservation of circadian clocks in marine zooplankton

**DOI:** 10.1093/nargab/lqad007

**Published:** 2023-01-31

**Authors:** Venket Raghavan, Gregor Eichele, Otto Larink, Eli Levy Karin, Johannes Söding

**Affiliations:** Quantitative and Computational Biology, Max Planck Institute for Multidisciplinary Sciences, Am Fassberg 11, 37077, Göttingen, Germany; Rhythms - Beating Cilia and Ticking Clocks, Max Planck Institute for Multidisciplinary Sciences, Am Fassberg 11, 37077, Göttingen, Germany; Evolutionary Biology, Zoological Institute, Technical University Braunschweig, Spielmannstraße 7, 38106, Braunschweig, Germany; Quantitative and Computational Biology, Max Planck Institute for Multidisciplinary Sciences, Am Fassberg 11, 37077, Göttingen, Germany; Quantitative and Computational Biology, Max Planck Institute for Multidisciplinary Sciences, Am Fassberg 11, 37077, Göttingen, Germany; Campus Institute Data Science (CIDAS), Georg-August-Universität Göttingen, Goldschmidtstraße 1, 37077, Göttingen, Germany

## Abstract

Zooplankton are important eukaryotic constituents of marine ecosystems characterized by limited motility in the water. These metazoans predominantly occupy intermediate trophic levels and energetically link primary producers to higher trophic levels. Through processes including diel vertical migration (DVM) and production of sinking pellets they also contribute to the biological carbon pump which regulates atmospheric CO_2_ levels. Despite their prominent role in marine ecosystems, and perhaps, because of their staggering diversity, much remains to be discovered about zooplankton biology. In particular, the circadian clock, which is known to affect important processes such as DVM has been characterized only in a handful of zooplankton species. We present annotated *de novo* assembled transcriptomes from a diverse, representative cohort of 17 marine zooplankton representing six phyla and eight classes. These transcriptomes represent the first sequencing data for a number of these species. Subsequently, using translated proteomes derived from this data, we demonstrate *in silico* the presence of orthologs to most core circadian clock proteins from model metazoans in all sequenced species. Our findings, bolstered by sequence searches against publicly available data, indicate that the molecular machinery underpinning endogenous circadian clocks is widespread and potentially well conserved across marine zooplankton taxa.

## INTRODUCTION

The zooplankton are a heterogeneous group of marine eukaryotes characterized by their free-floating nature and limited motility in the water column (1,2). They may be planktonic (free-floating) throughout their lifecycle (holoplankton) or only for a portion thereof (meroplankton). Zooplankton are typically classified on the basis of body size as microzooplankton (<200 μm) and mesozooplankton (>=200 μm) depending on whether or not they pass through a 200 μm filter screen (3,4). Zooplankton have a cosmopolitan biogeographic distribution and are taxonomically diverse. While the microzooplankton are predominantly protists, the larger mesozooplankton are predominantly metazoans. Although a species headcount is hard to estimate due to confounding factors such as cryptic species-level diversity (5) and in parts historical methodological constraints (6), the latest estimates from the MetaZooGene Barcode Atlas and Database indicate some 45,345 documented metazoan zooplankton species, of which 11,356 species have been identified on the basis of gold standard DNA barcodes, spanning 15 phyla and 41 orders/classes (7).

Zooplankton are important constituents of the multi-trophic marine food webs and play a fundamental role in sustaining the numerous ecosystem services provided by marine environments (8). For instance, many marine organisms of importance to human society (e.g., commercially valuable fish such as herrings and sardines) prey on zooplankton species (9). More importantly, marine zooplankton play a crucial role in the aquatic carbon cycle. Zooplankton are among the main predators/consumers of photoautotrophic phytoplankton (10,11) which consume and fix atmospheric carbon dioxide (CO_2_) photosynthetically. In addition to regulating phytoplankton populations (12,13), zooplankton predation upon phytoplankton directly contributes to the biological carbon pump (BCP): the process by which atmospheric carbon is sequestered in deep ocean waters (14). Namely, zooplankton help channel sequestered carbon in the form of ingested phytoplanktonic biomass deeper into the ocean through multiple processes (15) including diel vertical migration (DVM; migrations along the height of the water column with a period of approximately 24 hours) (16–19) and production of sinking fecal pellets (14,20). As the atmospheric concentrations of CO_2_ would be hundreds of times higher than its current value if the BCP were to be absent or impaired (21), marine zooplankton are directly implicated in climate regulation. Further, as marine zooplankton typically have short lifespans (22), changes in the abundance, distribution, and diversity of their populations arising from environmental and anthropogenic effects can be readily observed. As a result, zooplankton are useful as bioindicators to monitor the health and integrity of marine ecosystems (23).

Unfortunately, marine zooplantkon populations are being adversely affected by climate change and human interference, and the ecological consequences of damage to zooplankton is predicted to be severely negative. Among others, these include weakened trophic couplings (24), altered zooplankton community structure and composition (25,26), weakened carbon sequestration capability (27), and loss of oceanic oxygen (28). These consequences are hard to quantify, in part because of the unexpected ways in which stressors can affect zooplankton biology and behavior. For instance, work in the copepod *Centropages velificatus* has indicated that survivability and egg production decreased in response to moderate heat stress (29). In contrast, a recent study which simulated temperature adaptation in the brine shrimp *Artemia franciscana* indicated that these organisms developed phenotypic tolerance to elevated temperatures despite the absence of accompanying genetic and/or epigenetic marks (30), suggesting that there are potentially not only species-specific adaptations but also unexplored evolutionary effects at play. Therefore, it is of considerable interest to study and characterize the molecular-biological mechanisms that underpin zooplankton biology and behavior. In this regard, there is perhaps no molecular circuit that is as important as the circadian clock.

Circadian clocks facilitate the fundamental task of keeping time in biological systems. These intrinsic biomolecular pacemakers are characterized by two traits – the ability to self-sustain a period of circa 24 hours absent any external input and the ability to synchronize their oscillations with external cues using one or more input signals (31). This confers a powerful advantage (32–34) upon the host organism/cell as it is able to anticipate – and adapt to – periodic changes in its environment, by virtue of the numerous biological processes under circadian control (32,35,36). Much of our current understanding of circadian clocks stems from studies in two model organisms: the fruit fly (*Drosophila melanogaster*) and the house mouse (*Mus musculus*) (37). The general circadian mechanism here comprises a pair of interlaced transcription-translation feedback loops (TTFLs) that exert regulatory control through a set of transcription factors. The TTFLs ensure the rhythmic expression of the clock’s components as the genes of the transcription factors are themselves under the control of the clock. Regulatory control over other biological processes is exerted by means of a complement of so-called clock-controlled genes (CCGs) (35,38,39). Thereby, these CCGs directly link the molecular circadian clock to body physiology and behavior (40,41).

The fly and mouse clocks are mechanistically similar and consist of several homologous components (37). In *D. melanogaster*, the core TTFL consists of a pair of transcriptional activators and two transcriptional repressors. At dawn, nuclear concentrations of the activators Clock (CLK) (42) and Cycle (CYC) (43) gradually peak, favoring their heterodimerization and concomitant binding to E-boxes present in the regulatory regions of a number of genes (which thereby come under their control). Among these are the genes of the repressors Period (PER) and Timeless (TIM) (44). Subsequently, as a result of their transcription having been activated, the cytoplasmic concentrations of these repressors increase over the course of the day. In the night, PER and TIM heterodimerize and translocate into the nucleus. Here, they antagonize the activity of CLK-CYC by binding their complexes, leading to downregulation of the genes under their control. As a consequence, the concentrations of PER and TIM also diminish overnight, leading to the initiation of the next circadian cycle as CLK-CYC repression is gradually abolished towards dawn.

For unkonwn reasons, rhythmic expression of *Clk* is important for proper circadian functionality (37,45). It is controlled by a second TTFL that is itself under circadian control via CLK-CYC (46). This TTFL comprises Vrille (47) (VRI) and PAR (Proline and Acidic Rich) domain protein 1ε (PDP1ε; henceforth referred to as PDP1e) (46). Both proteins belong to the same basic leucine zipper (bZIP) transcription factor family, and compete for the V/P-boxes in the promoter region of *Clk*. VRI accumulates earlier and acts as a repressor of *Clk*. Later nuclear accumulation of PDP1e restores expression of the gene, thereby effectuating rhythmic expression of *Clk*. Additionally, in *D. melanogaster*, the light-sensitive cryptochrome CRY1 functions as an input pathway to the circadian clock by facilitating proteosomal degradation of TIM in a light-dependent maner (48,49). In other insects (and arthropods in general), CRY1 exists alongside its light-insensitive sibling CRY2 which is absent in *D. melanogaster* (50). CRY2 is a transcriptional repressor which takes the place of TIM in heterodimerization with PER, and is therefore a component of the core clock in these organisms (50).

The murine clockwork also consists of two TTFLs, and is largely homologous to its counterpart in the fly. The core TTFL is composed of homologs of fly CLK, CYC, PER, and (insect) CRY2 respectively (37,51). The second TTFL, however, differs from the one in Drosophila, and is composed of the paralogous activators REV-ERBa (Nuclear receptor subfamily 1 group D member 1/NR1D1) and REV-ERBb (Nuclear receptor subfamily 1 group D member 2/NR1D2), and the repressors retinoid-related orphan receptors RORα (NR1F1; hitherto referred to as RORa), RORβ (NR1F2), and RORγ (NR1F3) (52,53). By binding to the cognate enhancer elements, these proteins establish the rhythmic expression of *Cyc* (known as *Bmal1* in mice).

Although the precise evolutionary origins of circadian clocks in various phyla are unclear and debated (54,55), circadian clocks have been encountered in nearly every branch of the tree of life, with examples in heterotrophic and photoautotrophic bacteria (56,57), in fungi (58), and in plants (59,60). Needless to say, the circadian clock has also been encountered in metazoans wherein it appears to be highly conserved based on evidence from arthropods (45,61,62) and mammals (51,63). Mounting evidence – albeit inconclusive – has hinted at the existence of similar oscillatory timekeeping mechanisms in archaea (64,65).

As in other organisms, the circadian clocks in marine zooplankton play a central role in their biology, and they appear to be affected by environmental changes in unexpected ways. In *Daphnia pulex*, for example, it was demonstrated that the expression of the Period gene is suppressed in order to enhance salt tolerance caused by high levels of road salt, representing an unusual alteration to circadian functionality in response to environmental stress (66). Artifical light pollution also appears to affect the clock, with the expression of the Cryptochrome 2 (CRY2) gene being altered in *Daphnia magna* (with implications for growth and feeding) in response to exposure to artificial light at night (67). Altered circadian clock functionality can have potentially wide-ranging and far reaching consequences since zooplankton clocks have been implicated in influencing swimming behavior (68), feeding behavior (69), photoperiod-induced diapause (a survival strategy) (70), photoperiod adaptation (71), and, importantly, DVM (72,73).

Unfortunately, circadian clocks have been investigated in only a handful of zooplankton species. These happen to be predominantly arthopod crustaceans such as *Daphnia pulex* (74,75), *Daphnia magna* (70,71,76), *Calanus finmarchicus* (77), *Euphausia superba* (78), *Meganyctiphanes norvegica* (79), and *Jasus edwardsii* (80) to name a few; among non-crustacean zooplankton only model species such as the marine annelid *Platynereis dumerilii* have had their circadian clocks investigated (81). Characterization of the circadian clock in most zooplankton species has been hampered by inadequate sequencing, in particular, of transcriptomes which, when assembled *de novo*, can serve as inexpensive and accessible catalogs of expressed genes (82,83).

In this study, we used short-read RNA sequencing (RNA-seq) to assemble *de novo* and annotate the transcriptomes of a diverse set of marine zooplakton from six phyla: ten arthropod crustaceans, two annelids, two cnidarians, a phoronid (horseshoe worm), a chordate tunicate, and an echinoderm. Our transcriptomes are among the first sequencing data available for several of these marine zooplankton species. Subsequently, we demonstrate the presence of orthologs to canonical metazoan circadian clock components CLK, CYC, PER, TIM, CRY1 from *D. melanogaster*, CRY2 from *Danaus plexippus* (the monarch butterfly), and REV-ERBa and RORa from *M. musculus* among protein sequences derived from these assemblies. In addition to considerably expanding sequencing data from wildtype zooplankton, our results suggest that most if not all zooplankton species may use internal circadian clocks composed of components homologous to canonical counterparts found in model metazoans to control their biology and behavior.

## MATERIALS AND METHODS

### Sampling and RNA sequencing

Samples were obtained from the Helgoland Roads site (84) (latitude: 54.1883, longitude: 7.9000) in the North Sea during a routine expedition by the station ship *Aade* (https://www.awi.de/en/expedition/research-vessel-and-cutter/more-ships.html) in late 2018 (see Figure 2 for sampling dates). The samples of *Acartia clausii* and *Acartia tonsa* were acquired at a later date from the standing culture stock of the Alfred-Wegener-Institute, Helmholtz-Center for Polar and Marine Research (Helgoland, Germany). Marine microorganisms were captured en masse using a Hensen net (85,86) with a mesh size of 250 μm. All samples were collected during the early morning hours (but at varying times) from a depth of 15 m. The captured organisms were maintained in 400 ml fresh sea water at 6°C in darkness until further processing (which took place on the day of capture, or the day after in some cases). A first round of handpicking under stereo microscopy was used to sort and identify various copepods and other planktonic species of interest. A subsequent round of handpicking yielded between 1 and 50 individuals per species which were then used for sequencing. All selected individuals were approximately between 300 μm–1 mm in size. These were suspended in 1.5 ml Eppendorf tubes containing 50 μl fresh sea water to which 500 μl of cold TRIzol^TM^ (Invitrogen, Thermo Fisher Scientific, Germany) was added and vigorously shaken. The samples were stored at -60 °C for transport to the mainland.

RNA isolation and sequencing was performed at the NGS Integrative Genomics Core Unit, Institute for Human Genetics, University Medicine Göttingen (Germany). RNA was isolated using TRIzol^TM^ (Invitrogen, Thermo Fisher Scientific, Germany) with the addition of 0.1 mg/ml glycogen (Sigma-Aldrich, Germany). RNA-seq libraries were prepared using the TruSeq mRNA Library Prep Kit (Illumina, USA). DNA fragment length distributions were measured on a Fragment Analyzer (Advanced Analytical/Agilent, USA) prior to pooling. Sequencing was performed on an Illumina HiSeq 2500 sequencer (Illumina, USA) with the 2 × 250 base pairs and paired-end option. The raw sequencing data were de-multiplexed using bcl2fastq v2.17.1.14.

To visualize the diversity of the sequenced samples, we derived a taxonomic tree using NCBI taxonomy identifiers as inputs to the taxize (87,88) R (89) package. For samples with no available taxonomy identifiers, the identifier of a sister species was used as a stand-in to construct the tree (with the tip label in the tree being replaced with the name of the sample’s species). The tree itself was then visualized using the ggtree (90) R package. A script automating this procedure can be found in data and code repository accompanying this publication (see Section ‘Data and code availability).

### *In silico* workflow overview

**Figure 1.**
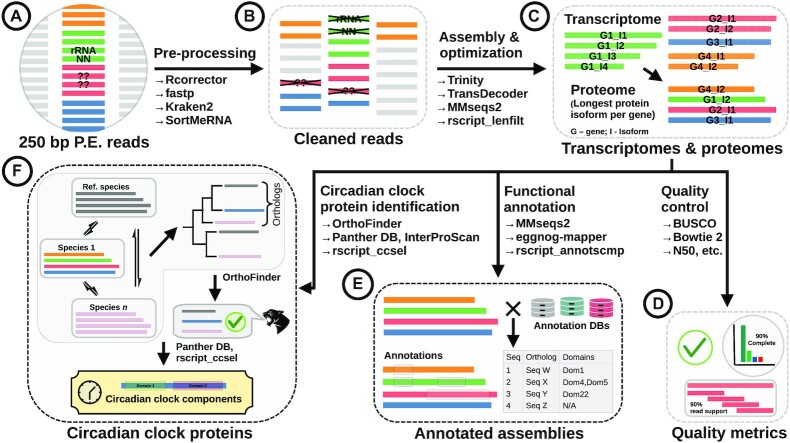
Bioinformatics workflow used to assemble *de novo* and annotate transcriptomes of the marine zooplankton, and subsequently identify circadian clock protein candidates from translated protein sequences. Black solid arrows indicate the direction of data flow. Text adjacent to arrows indicate an action (e.g., assembly) and the tooling used to achieve it (e.g., Trinity for assembly). Tools with ‘rscript’ in their names are custom scripts written in the R programming language. For details see Section ‘Methods’. P.E.: paired-end. Colors of the horizontal bars in figure sections (**A**)–(**F**) indicate different genes. In figure subsection (**C**) the multiple horizontal bars grouped together (top half of the figure subsection) are translated protein sequences of transcript isoforms originating from the same gene; in the bottom half of that subsection only the protein sequences of the selected isoforms (one per gene) are indicated. Figure subsection (**F**) retains this coloring scheme for the protein sequences of one input species (Species 1) as an example for the process described by it, namely clock protein identification by means of orthology to a known clock protein sequence from a reference proteome; here sequence sets representing the other species are not colored in this manner, and are instead colored uniformly with a single color.

We developed a robust bioinformatics workflow to achieve the multiple goals of high quality *de novo* transcriptome assembly, annotation, and circadian clock sequence identification (Figure 1). Paired-end RNA-Seq data were obtained as detailed in Section ‘Specimen acquisition and RNA sequencing’ (Figure 1(A)) and pre-processed using tooling described in Section ‘RNA-seq data pre-processing’ (Figure 1(B)) to retain high quality reads only. The reads were assembled *de novo* to obtain initial transcriptomes that were then optimized to yield condensed-but-representative ‘final’ transcriptomes (and accompanying protein sequence sets/proteomes) as described in Section ‘*De novo* transcriptome assembly and optimization’ (Figure 1(C)). Select quality control metrics were evaluated before and after optimization to validate the procedure and evaluate the quality of data (Figure 1(D)). The proteomes were then used to acquire functional annotations against a select set of databases (Section ‘Functional annotation’ and Figure 1(E)). Finally, a phylogeny-based approach was used to identify circadian clock proteins in the sequenced species based on pairwise orthology to known circadian clock protein sequences (Section ‘Identification of circadian clock proteins’; Figure 1(F)).

**Figure 2.**
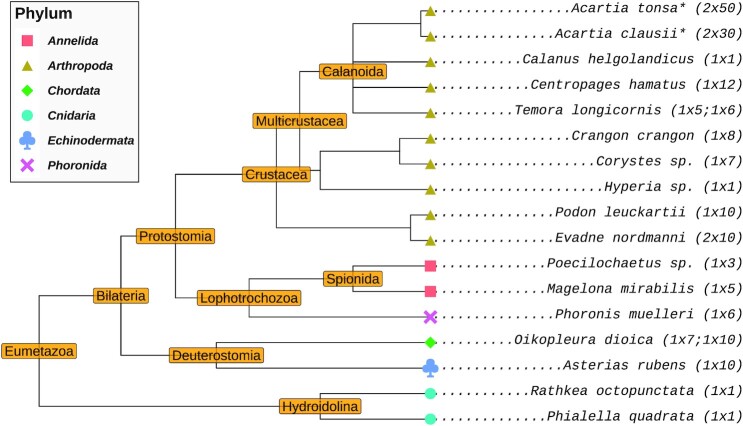
Taxonomic tree of the species investigated in this study. Species marked with an asterisk were sampled on 30.07.2020. All other species were sampled on 21.06.2018. Values indicated in parentheses are the number of RNA-seq data sets and the number of individuals sacrificed for those data sets, respectively.

### RNA-seq data pre-processing

The tool Rcorrector (91) was used to detect and repair reads containing sequencing errors. The data were then processed with fastp v0.20.1 (92) to remove reads with ambiguous (‘N’) base calls, trim adapter sequences, and eliminate reads with low average quality scores (PHRED score < 20). Both reads (forward and reverse) were discarded even when only one of the reads was affected, as we did not wish to retain unpaired reads for assembly. Subsequently, the metagenomic read classifier Kraken2 v2.1.2 (93) was used to detect and exclude read pairs originating from ‘contaminant’ sources. These included bacteria, archaea, viruses, plasmids, humans, vector constructs (UniVec_Core), fungi, protozoans, and plants (as defined by the PlusPFP Kraken2 reference data set available at https://benlangmead.github.io/aws-indexes/k2). This step was especially important as zooplankton commonly feed on cyanobacteria and other prokaryotes (11,94,95), and was possible for contaminants to infiltrate the data. Although the poly-A selection by poly-T priming strongly depletes non-messenger RNAs, rRNAs can still contaminate the library (96). Therefore, SortMeRNA v4.3.0 (97) was used to eliminate residual rRNA and tRNA in the data by mapping the reads against the included smr_v4.3_sensitive_db_rfam_seeds.fasta database. The reads were quality controlled before and after each of these filtering steps using FastQC v0.11.9 (98). Samples with multiple sequencing data sets (see Figure 2) were pooled prior to the aforementioned steps in order to obtain a single assembly per species.

### *De novo* transcriptome assembly and optimization

The filtered reads were assembled *de novo* with Trinity v2.11.0 (99,100) using default parameters. The assembled transcripts were filtered using SeqKit v0.14.0 (101) to exclude transcripts shorter than 200 nucleotides. Assembly quality was assessed using two independent metrics. First, the read support for the assemblies – the percentage of reads mapping back to the sequence sets – was estimated using Bowtie2 v2.4.2 (102). Second, BUSCO v4.1.4 (103) (Benchmarking Universal Single-Copy Orthologs) was used to assess the presence of orthologs to sets of universal single-copy genes for Arthropoda (arthropoda_odb10; 1013 genes) and Eukaryota (eukaryota_odb10; 255 genes) from OrthoDB v10 (104). This offers an indication of the transcriptome’s completeness: the aforementioned single-copy genes are universal and believed to be constitutively expressed, and therefore a high-quality assembly should be able to furnish orthologs to a large proportion of these sequences. TransDecoder v5.5.0 (https://github.com/TransDecoder/TransDecoder) was used to predict and translate coding sequences in the assemblies. The –single_best_only option was supplied to retain only the best scoring open reading frame (ORF) for each transcript. The resulting protein sequence sets (proteomes, hereafter) were used for all downstream procedures as sequence comparisons with amino acid sequences are more sensitive in comparison to nucleotide sequences (105).

A two-step strategy was adopted to reduce redundancy in the data and obtain final, representative proteomes (and corresponding transcriptomes). First we used MMseqs2 v12.113e3 (106) to cluster the transcriptomes at 98% minimum sequence identity and target sequence coverage to eliminate nearly identical sequences; the longest sequence from each cluster was retained as the representative. Trinity conveniently groups the assembled sequences into groups of transcript isoforms (that all hypothetically originate from the same gene) (100). We used this information to reduce the redundancy in the assemblies even further by retaining only the longest protein isoform as the representative for each Trinity gene cluster using a custom R script (89) (rscript_lenfilt.R). We monitored BUSCO scores and read support to assess the redundancy reduction and completeness of the resultant proteomes. We constructed the final, redundancy-reduced representative transcriptomes by extracting the nucleotide sequence(s) corresponding to the selected protein sequence isoforms from the initial Trinity assemblies.

### Transcriptome functional annotation

The translated protein sequences were annotated by searching through the UniProt/Swiss-Prot (107) database (release 2021_03) using MMseqs2 v12.113e3 with an E-value cut-off of 10^−5^ and the highest search sensitivity available in the tool (-s 7.5). The match with the lowest E-value was retained as the definitive homolog for each query sequence. The protein sequences were also annotated using eggnog-mapper v2.1.3 (108,109) against the EggNOG database (110) to infer functionally similar orthologs and obtain gene ontology (GO) annotations (111,112). The taxonomic scope of the searches was limited to Metazoa using the –tax_scope Metazoa argument. The –pfam_realign realign argument was supplied to obtain Pfam domain annotations (113). The UniProt/Swiss-Prot and eggnog-mapper results were merged using a custom R script (rscript_annotscmp_mainjob.R) to generate the final annotations for each data set.

### Identification of circadian clock proteins

We reasoned that the circadian systems of our target species must have functioning orthologs to known circadian clock proteins from insects (in specific, arthropods) and mammals, given the rather widespread conservation of the core components of this circadian model among metazoan species. Therefore, we decided to use well-characterized circadian clock proteins from model organisms as ‘baits/references’ to seek out candidates from the assemblies on the basis of homology. To establish a non-redundant catalog of baits, we investigated the circadian systems of the fruit fly (*D. melanogaster*) and mouse (*M. musculus*) which, taken together, can be considered to be representative of the circadian systems of metazoans. We elected to use the *D. melanogaster* orthlogs for the core components CLK, CYC, PER, TIM, CRY1, VRI, and PDP1e respectively. As the fly does not possess a CRY2 (a core clock component in other insects (45,62)), the well-investigated CRY2 from the monarch butterfly (*D. plexippus*) was also included as a bait (114). Although the mouse and fly circadian clocks (representing the more general mammalian and insect clocks respectively) are largely similar, with most core components being orthologous, some differences still exist. In particular, only two components of the second TTFL in the mouse clock are known to have orthologs in insects that are involved in the corresponding circadian clocks. These are E75 (ortholog of mouse REV-ERBa) and HR3 (ortholog of mouse RORa) respectively, whose genes and gene products have been implicated in clock functionality in non-model insects such as *Thermobia domestica* and *Gryllus bimaculatus* (115,116). Therefore REV-ERBa and RORa from mouse were included as baits also to complete the ‘metazoan-representative’ circadian clock protein catalog. The UniProt identifiers for the baits can be found in Table 2.

As our approach required whole proteomes as inputs (see below), reference proteomes consisting of one protein sequence per gene were obtained from UniProt (release 2021_03) for *Drosophila melanogaster*, *Danaus plexippus*, and *Mus musculus* by downloading the associated protein data sets using the ‘Download one protein sequence per gene (FASTA)’ option from corresponding UniProt Proteomes webpages. This ensured that each protein (especially the circadian clock proteins) was represented by one and one sequence only as this could have potentially confounded orthology assignments used within our workflow (UniProt is known to have duplicate sequences despite strict curation). The circadian clock proteins of interest present in these proteomes were identified and highlighted by appending uniquely identifiable strings (namely ‘SOIREF’) to their FASTA headers to be used as baits; this permitted easy identification and extraction of the relevant baits and their orthologs within our processing scripts.

Our approach to identifying circadian clock protein candidates differed significantly from previous work with similar objectives. Studies have traditionally relied on sequence similarity searches to identify candidates, with a well-characterized clock protein serving as the query and the transcriptome/proteome sequence set as the target(s) (e.g., Christie et al. (80)); the best match, or all matches with the lowest e-value, were then extracted as candidates. Here, instead, we opted to take full advantage of the many proteomes at our disposal to identify candidate circadian clock proteins by pairwise orthology. To this end the assembled proteomes as well as the three reference proteomes were supplied as inputs to the comparative genomics tool OrthoFinder v2.5.2 (117). Shortly, OrthoFinder first performs exhaustive all vs. all searches to construct orthogroups (collections of homologs, essentially) which are then supplied to a novel algorithm to create rooted gene trees; these gene trees are then examined to automatically infer all possible sets of pairwise orthologs (and thereby, implicitly, also paralogs) within that gene family. To correctly root the gene trees, OrthoFinder can automatically infer a species tree by examining the input proteomes. Although the orthogroup (collections of orthologs) identification is apparently relatively robust to inaccuracies in this automatically-inferred species tree, the pairwise ortholog detection step is not. Therefore, to ensure maximum tool sensitivity, a species tree consisting of the input organisms (the 17 sequenced species and the three reference species) was constructed using NCBI taxonomic identifiers (118) with the taxize (87) and ape (119) R packages. For some sequenced samples, an identification down to the species level was unfortunately not possible despite best efforts (all samples were identified at least down to the genus). In these cases, the closest identified relative (another species from the same genus) was used as a stand-in for leaf in the species tree supplied to OrthoFinder.

OrthoFinder’s output conveniently tabulates all pairwise orthologs found between all possible pairs of input sequence sets. Candidates were extracted from this table by seeking out all identified pairwise orthologs to the bait sequences embedded in the reference proteomes by searching with the unique identifier strings assigned to them previously. These candidates were then vetted further by investigating their protein family (and sub-family) affiliations and interrogating for shared domains between the candidates and the corresponding pairwise baits. Evolutionary family affiliations were annotated using the PANTHER v15.0 (120) evolutionary and functional classification system. Domains were annotated against Pfam v33.1 (113); both PANTHER and Pfam were accessed via InterProScan v5.51-85.0 (121)). Annotatations were acquired for both the baits and the candidates, independent of the functional annotations described earlier. The candidates were subsequently vetted as follows. First, any sequence that did not receive any annotations from both PANTHER and Pfam databases were discarded. Then, candidates were discarded if they did not share at least one Pfam domain in common with the corresponding bait sequence. PANTHER assigns sequences to protein families and, if possible, sub-families within these families. To cull the candidate pool further, candidates that did not belong to the same PANTHER family as their bait were discarded. At this stage, each circadian clock component had multiple candidate sequences in some samples. In these cases, if candidates with the same sub-family affiliation as the bait were present, only these were retained (if such candidates did not exist, all candidates were retained for that clock component from that species).

This yielded a final set of high-confidence circadian clock candidate proteins. For these sequences we examined the Swiss-Prot homolog assignments from the functional annotation step were to confirm that the best match homolog was a circadian clock protein (or closely related member from the same family). All sequences cleared by these filtering stages were retained as potential candidates, even when multiple competing candidates were available for clock components that are known to be single copy. This had to be done as no reliable *in silico* methods were available to distinguish true-positive candidates (that are biologically active and functional) from false-positive ones, especially in the case of candidates that were not full-length (i.e., missing the start codon, stop codon, or both). In order to visualize the domain compositions and structures of the identified candidates, the sequences were annotated separately against the Pfam, SMART v7.1 (122), and CDD v3.18 (123) databases using InterProScan. These annotations were subsequently visualized as domain structure diagrams using seqvisr v0.2.3 (124). Multiple sequence alignments (MSAs), wherever necessary, were constructed with Clustal Omega (125) accessed via the msa (126) R package. Pairwise alignments were constructed using the Biostrings (127) R package.

### Additional sequence searches against NCBI data

We first identified all available transcriptome and genome assemblies on NCBI using the appropriate taxonomic identifiers for our species with Entrez esearch (128), and downloaded them using fastq-dump from the SRA ToolKit (https://github.com/ncbi/sra-tools). We the searched against these assemblies with MMseqs2 using the baits from *D. melanogaster*, *D. plexippus*, and *M. musculus* as queries. For this procedure, we retained the same search parameters used during functional annotation step; however, in this instance, we excluded all hits with query and target sequence coverage values below 20%. From these searches, we identified the sequence with the lowest e-value as the potential candidate. Even when multiple transcriptome and/or genome assemblies were available, we retained only a single sequence as the final candidate for each species against the corresponding query. These searches were performed only for those clock proteins for which the OrthoFinder-based workflow described above failed to produce a candidate. The sequences found here were not subjected to annotation, visualization, or detailed analysis in the manner the sequences from our workflow were. The accessions of the assemblies used for this search can be found in the supplements (supplementary file s8_cc_ncbicomp_accessions.txt).

## RESULTS AND DISCUSSION

### Sequencing and *de novo* transcriptome assembly

We sampled zooplankton from the Helgoland Roads (84), assigned taxa based on expert assessment of morphological and physiological features under stereo microscopy, and sequenced them in batches of 1 to 50 conspecific individuals (see Section ‘Methods’). Despite best efforts, three sets of organisms could not be identified down to the species level, and these have been denoted here using their genera: *Corystes sp*., *Hyperia sp*., and *Poecilochaetussp*.. The taxonomy of the 17 identified taxa shows their broad diversity, with 10 crustacean arthropods and seven species from five other metazoan phyla (Figure 2).

We sequenced a single paired-end RNA-Seq library for each species, with the exception of *Acartia clausii*, *Acartia tonsa*, *Evadne nordmanni*, *Oikopleura dioica*, and *Temora longicornis*, for which we pooled data from two paired-end libraries each. Sequencing yielded an average of 25 million paired-end reads per species which was reduced to an average of 20 million reads after pre-assembly processing (see Section ‘Methods’ for details). For most samples, over 80% of the reads were retained after processing (Table 1). Only the *A. clausii* and *A. tonsa* samples lost a large fraction of the reads (46.2% and 35.4% respectively), presumably due to a large number of ambiguous (N) base calls and other irreparable errors embedded in the sequencing output.

**Table 1.** Read counts and assembly statistics for the *de novo* transcriptome assemblies

		Number of reads		Transcripts in assembly		N50	
Organism	Phylum	Raw	Processed	Initial	Final	N50 (initial)	N50 (final)
*Acartia tonsa*	Ar	46728568	30183880 (64.6)	160483	22787 (14.2; 42.7*)	1452	1959
*Acartia clausii*	Ar	42990921	23138097 (53.8)	159937	23313 (14.6; 38.7*)	1377	1897
*Calanus helgolandicus*	Ar	19503965	17217713 (88.3)	164611	43040 (26.1; 30.2*)	1366	1570
*Centropages hamatus*	Ar	21834660	18449434 (84.5)	161672	23278 (14.4; 38.8*)	1573	2042
*Temora longicornis*	Ar	38035737	32327268 (85)	204478	25724 (12.6; 34.3*)	1611	2021
*Crangon crangon*	Ar	19496190	16749098 (85.9)	182017	22686 (12.5; 43.1*)	2340	2968
*Corystes sp*.	Ar	20596859	17303099 (84)	176664	28738 (16.3; 28.6*)	1912	2263
*Hyperia sp*.	Ar	14434862	12339911 (85.5)	127319	17429 (13.7; 21.3*)	1021	1408
*Podon leuckartii*	Ar	20965748	18577507 (88.6)	111883	21961 (19.6; 53.5*)	2535	2477
*Evadne nordmanni*	Ar	38147607	32851241 (86.1)	125959	14391 (11.4; 51.5*)	2861	2935
*Poecilochaetus sp*.	An	19082838	16260294 (85.2)	272460	35984 (13.2; 21.2*)	1325	1807
*Magelona mirabilis*	An	16287463	13439540 (82.5)	246407	27690 (11.2; 29.1*)	1358	2029
*Phoronis muelleri*	Ph	18102502	16002988 (88.4)	169860	24094 (14.2; 39.8*)	2000	2532
*Oikopleura dioica*	Ch	34115579	29875095 (87.6)	113214	19750 (17.4; 49.5*)	2275	2286
*Asterias rubens*	Ec	19841332	16895994 (85.2)	127179	17918 (14.1; 33.7*)	1881	2520
*Rathkea octopunctata*	Cn	17342954	15271220 (88.1)	77799	16332 (21; 61.5*)	2160	2574
*Phialella quadrata*	Cn	19314077	17345509 (89.8)	77732	16145 (20.8; 61.6*)	2338	2626

‘Initial’ and ‘Final’ refer to the status of the transcriptome before and after being subjected to an assembly optimization workflow. Numbers in parentheses indicate percentage value with respect to initial/raw numbers; the numbers in the parentheses marked with an asterisk under the column ‘Final’ in ‘Transcripts in assembly’ indicate the percentage of sequences in the final assembly that were identified as being complete open reading frames. N50: contigs of length greater than or equal to the N50 value contain 50% of all bases in the transcriptome. Phylum abbreviations: Ar - Arthropoda, An - Annelida, Ph - Phoronida, Ch - Chordata, Ec - Echinodermata, Cn - Cnidaria. nt: nucleotides

**Table 2.** Workflow validation: circadian clock protein orthologs from the reference proteomes to the designated bait/reference proteins, found using OrthoFinder

Reference	Ref. proteome match
**Arthropod**	
CLK (Dm O61735)	Dp: CLK (A0A212EGJ4), Mm: NOT FOUND
CYC (Dm O61734)	Dp: CYC (A0A212EKE6)
	Mm: ARNTL (Q9WTL8), ARNTL2 (Q2VPD4)
TIM (Dm P49021)	Dp: TIM (A0A212ETU4)
PER (Dm P07663)	Dp: PER (A0A212F9R2)
	Mm: PER1 (O35973), PER2 (O54943), PER3 (O70361)
CRY1 (Dm O77059)	Dp: CRY1 (A0A212EI23)
CRY2 (Dp A0A212FAM3)	Mm: CRY1 (P97784), CRY2 (Q9R194)
PDP1e (Dm Q8SZT1)	Mm: DBP (Q60925), HLF (Q8BW74), TEF (Q9JLC6)
VRI (Dm Q9VMS4)	Mm: NFIL3 (O08750)
**Mammalian**	
REV-ERBa (Mm Q3UV55)	Dm: EIP75B (P13055)
RORa (Mm P51448)	Dm: HR3 (P31396)

Indicated in the left column are the baits, and in the right their counterparts. Alongside the protein name of the bait and the source organism, the sequence’s UniProt identifier is also indicated. The baits are grouped by the phylogenetic clade they originate from: ‘Arthropod’ baits were all from *Drosophila melanogaster*, except for CRY2 (from *Danaus plexippus*); ‘Mammalian’ baits were from *Mus musculus*. Only one ortholog – CLK from *M. musculus* (O08785) – was not found. Species: Dm - *Drosophila melanogaster*, Dp - *Danaus plexippus*, Mm - *Mus musculus*. Protein names: CLK (Circadian locomotor output cycles kaput), CYC (Cycle), PER (period), TIM (Timeless), CRY1 (Cryptochrome 1), CRY2 (Cryptochrome 2), PDP1e (PAR Domain Protein 1 epsilon), VRI (Vrille), REV-ERBa (NR1D1/Nuclear Receptor Subfamily 1 Group D Member 1), RORa (RAR Related Orphan Receptor A; NR1F1/Nuclear receptor subfamily 1 group F member 1), NPAS2 (Neuronal PAS Domain Protein 2), ARNTL (Aryl hydrocarbon Receptor Nuclear Translocator-Like protein), DBP (D-box Binding Protein), HLF (Hepatic Leukemia Factor), TEF (Thyrotroph Embryonic Factor), NFIL3 (Nuclear Factor, Interleukin 3 Regulated), EIP75B (Ecdysone-induced protein 75B, isoforms C/D; NR1D3), HR3 (Probable nuclear hormone receptor HR3). PER1, PER2, and PER3 are in-paralogs. ARNTL1 and ARNTL2 are in-paralogs. Note: these orthologs were not used as baits themselves (see text for clarification)

**Figure 3.**
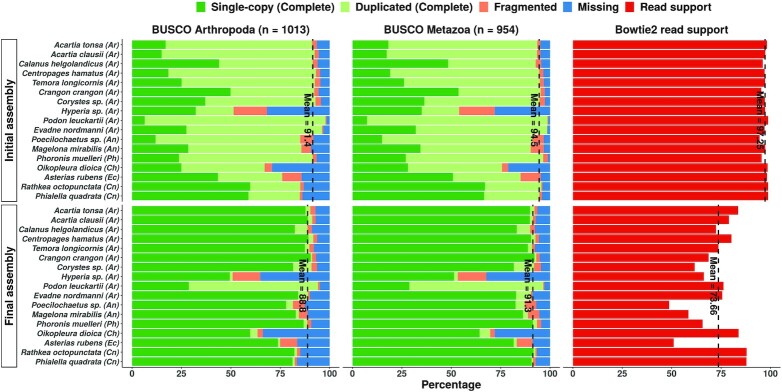
Quality assessment statistics for the initial (top) and final (bottom panels) *de novo* transcriptome assemblies. The vertical dashed lines indicate the mean completeness levels (single-copy + duplicated) in the BUSCO panels, and the mean read support across all assemblies in the Bowtie2 panels. Initial and final refer to the status of the assemblies prior to – and after – an assembly optimization process. Phylum affiliations are indicated in parentheses after the species name. Phylum abbreviations: Ar - Arthropoda, An - Annelida, Ph - Phoronida, Ch - Chordata, Ec - Echinodermata, Cn - Cnidaria.

*De novo* assembly with Trinity v2.11.0 (99) yielded ‘initial’ transcriptomes with about 156,000 transcripts on average, and a minimum length of 200 nt (Table 1). The cnidarian *Phialella quadrata* had the smallest assembly (77,732 transcripts), while the annelid *Poecilochaetus sp*. had the largest (272,460). Quality control metrics indicated that all initial assemblies were of good quality (Figure 3, Table 1). We first assessed the N50 metrics for the assemblies. The N50 value is that length at which 50% of the bases in the assembly are incorporated into contigs (contigous sequences resulting from assembly of short reads) of this length or greater. The N50 is a good proxy for assembly quality as bad assemblies will yield predominantly short sequences which would in turn depress this metric. N50 assembly quality values ranged from 1021 nt (the arthropod *Hyperia sp*.) to 2861 nt for the arthropod *Evadne nordmanni*. The mean N50 for these assemblies was 2230 nt (Table 1). Similarly, a very high percentage of paired-end reads were mapped back to the respective assembly by Bowtie2 (102) (mean = 97.25%), indicating that most of the sequenced data was informative for the construction of transcripts (top panels, Figure 3). We also checked for the presence of universally conserved single copy genes using BUSCO (103) to gauge the completeness of the sequencing and assembly processes. With a mean completeness value of 94.50% against the metazoan benchmark data set, our assemblies were highly complete (top panels, Figure 3).

*De novo* assembled transcriptomes are very redundant due to the presence of transcript isoforms arising from sequencing pre-mRNAs and from physiological and noise-related alternative splicing events (129). Redundancy may also be the result of closely related paralogs and/or genetic variability confounding the assembler. To this end, we implemented an assembly optimization workflow to obtain representative but non-redundant assemblies for downstream analyses and subsequent publication (see Section ‘Methods’). The optimization strategy depended on translating the sets of transcripts to obtain sets of predicted protein sequences (henceforth referred to as proteomes), and selecting the longest protein sequence – and corresponding transcript – for each transcript-isoform cluster to obtain a representative subset of sequences. Having implemented this workflow, we compared the quality of the initial (un-optimized) and final (optimized) assemblies to gauge its effectiveness. The transcript counts and N50 values for the final assemblies are indicated in Table 1 (columns 5 and 7 respectively).

With an average size of 23,604 transcripts, the optimization workflow produced final non-redundant assemblies vastly condensed in size (Table 1). In contrast, the N50 values improved across the board, increasing from a mean value of 1846 nt to 2230 nt (Table 1). Despite retaining only 15.72% of the original transcripts on average, these final assemblies were nevertheless nearly as complete (91.30% average completeness), as attested by their BUSCO scores against the Metazoa data set (bottom panels, Figure 3). We also evaluated all assemblies against the OrthoDB v10 (104) *Arthropoda*BUSCO data set as we considered the *Metazoa* data set to be too non-specific and not necessarily indicative of assembly quality on its own, and because a majority of our samples were arthropods or arthropod-adjacent species (Figure 2). The completeness trends observed earlier were replicated here: the average completeness across the final assemblies was 88.80% as opposed to 91.40% for the initial assemblies (against the arthropod BUSCO data set). While the non-arthropod species – somewhat unsurprisingly – did obtain lower completeness scores, the reduction in completeness scores between the initial and final assemblies was small; an acceptable compromise considering the relatively large reduction in redundancy. For instance, in comparison to the initial assembly of the tunicate *Oikopleura dioica* (67.10% completeness), the corresponding final assembly’s redundancy is almost negligible while still being 63.50% complete according to BUSCO (against the arthropod BUSCO data set). Finally, although the proportion of ‘complete’ ORFs (featuring a start and stop codon) as predicted by TransDecoder was only 39.9% on average, we note that the tool can, on occasion, predict a longer ‘incomplete’ ORF that essentially encapsulates the actual (and complete) ORF.

Differences in read support between the initial and final assemblies were more pronounced. The initial assemblies had extremely high read support values across the board (averaging 97.25%) highlighting Trinity’s prowess in extensively recovering transcript isoforms even from small data sets. Naturally, these assemblies were highly redundant, as evidenced by their BUSCO duplication scores (Figure 3). Our filtering procedure compacted the assemblies significantly, as the average read support dropped to 73.66%. In some cases, such as that of *Poecilochaetus sp*., the final assembly seems to comprise of transcripts derived from only about half the available reads (Figure 3). Further, the multi-mapping rate (proportion of reads that were mapped to more than one transcript) dropped significantly. For instance, the final assembly for *Calanus helgolandicus* has a multi-mapping rate of only 12.04% as opposed to 66.68% for the initial assembly. The final assemblies thus obtained were compact (as evidenced by the read counts) but nevertheless still representative as they were complete (as evidenced by the BUSCO scores) and non-redundant (as evidenced by the reduction in multi-mapping rate).

Finally, we note that two assemblies were of poorer quality in comparison to the others. The assembly of *Hyperia sp*. was quite incomplete, even when evaluated against the Eukaryota BUSCO data set which comprises of fewer genes than the Arthropoda data set. It also obtained the lowest N50 value in the cohort (1048 nt). The closest related species in our data set (*C. crangon* and the unidentified *Corystes sp*.; Figure 2) produced noticeably better assemblies in comparison with better BUSCO scores. Taken together, sequencing of this unidentified crustacean yielded a very fragmented assembly. We assume that this could be attributed to the low sequencing depth (14.43 million reads; Table 1) and the fact that the RNA was extracted from the tissue of a single individual and not from a pool of multiple individuals as was the case with other organisms in this data set. The other problematic sample was the crustacean *Podon leuckartii* whose assembly appeared to be highly redundant even after optimization according to its BUSCO scores (Figure 3). However, this assembly did not have a high multi-mapping read rate which indicates the proportion of reads which are shared by more than one assembled contig. The multi-mapping read rate was only 3.10% in the final assembly, versus an initial multi-mapping rate of 66.72% (data not shown). The closest relative to *P. leuckartii* in our data set is *Evadne nordmanni*, and its assembly does not share these anomalous characteristics. We therefore speculate that the apparent redundancy could be caused by ubiquitous tandem gene clusters resulting from an elevated gene duplication rate as observed in other crustaceans such as *Daphnia pulex* (130).

In comparison to other studies that have performed *de novo* transcriptome assembly with similar organisms (for instance (131)), we worked with significantly smaller RNA-Seq data sets comprising of ca. 20 million reads on average. Despite this handicap we managed to produce high quality assemblies and functional annotations for most of the sequenced species. Transcriptome assemblies (and associated assembly metrics) were available from literature for a handful of species for comparison. In general, our N50 values were comparable to or better than values reported in literature. For instance, Semmouri et al. (132) report an N50 of 694 nt for a *Temora longicornis* assembly with 179,569 transcripts. Our final assembly for the same organism produced an N50 of 2021 nt. In comparison to their reported BUSCO completeness score of 54%, our assembly fairs significantly better (90.9%). Similarly, Asai et al. (95) report a N50 value of 1784 nt for a *Calanus helgolandicus* assembly consisting of 30,339 transcripts, while we obtained a N50 of 1570 nt and 43,040 transcripts. Zhao et al. (131) assembled 113,786 transcripts with an N50 of 1665 nt for *A. tonsa*, while we assembled 160,483 transcripts with an N50 of 1959 nt (Table 1). This publication did not perform a BUSCO assessment, so we were unable to compare assembly completeness. It must be noted that the Semmouri et al. (132) and Zhou et al. (131) publications did not attempt to optimize/redundancy-reduce their *de novo* assemblies as performed in our study. In contrast Asai et al. (95) did filter their assembly aggressively by retaining only the longest transcripts per Trinity gene cluster and imposing a minimum expression threshold of 1 > read per kilobase per million mapped reads (RPKM) in at least two samples. We presume that their N50 values are more comparable to the ones reported here as a consequence of this strategy. We were unable to ascertain how their filtering approach affected assembly completeness as BUSCO scores were not reported in the publication.

### Transcriptome functional annotation

Functional annotations attach human-comprehensible identifiers to the otherwise intractable sequencing data, and represent an important aspect of making the data analyzable (83). Annotations are especially useful for *de novo* assembled transcriptomes as the sequences are completely unidentified otherwise, and more importantly, often represent the first tranches of sequencing data from the source organisms (as is the case here). Of the 17 species we sequenced, only 7 had BioProject identifiers suggesting availability of genomes or transcriptomes on NCBI; even among these, there were instances where we found only the raw sequencing reads on NCBI and no deposited assembly. Likewise, many species from our study do not even have any amino acid or nucleotide sequences deposited in RefSeq (see supplementary file s2_ncbi_assem_counts.csv). Organisms such as *Podon leuckartii* did not have any publicly available sequence data whatsoever as of writing. In light of this, we elected to provide functional annotations for all assembled transcriptomes.

We used the proteomes produced by our optimization pipeline for annotation as most functional information is only meaningful in the context of protein sequences (e.g., domains). The protein sequences were searched against Swiss-Prot (107) using MMseqs2 (106) to identify homologs with high quality annotations. We used a very stringent E-value cut-off of 10^−5^ to minimize false positive homolog assignments. We did not impose a coverage cut-off to ensure that all sequences irrespective of their length would receive annotations. eggnog-mapper (108,109) was used to infer orthologs from the EggNOG (110) database, and to annotate GO (gene ontology) terms (111) and Pfam (113) functional domains.

**Figure 4.**
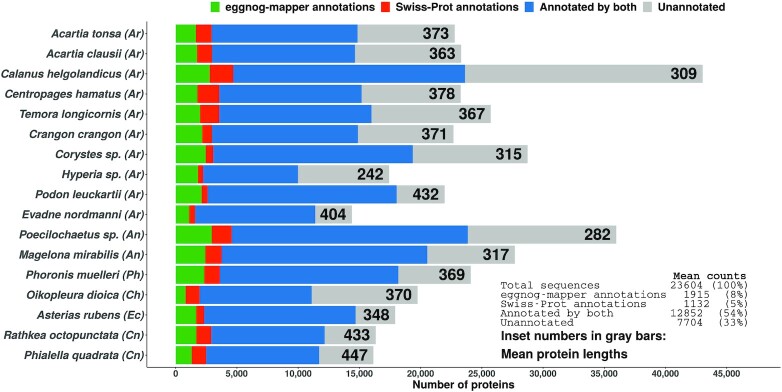
Sequence annotation statistics for the *de novo* assembled transcriptomes. The inset table refers to aggregated sequences from all transcriptomes. Phylum affiliations are indicated in parentheses after the species name. Phylum abbreviations: Ar - Arthropoda, An - Annelida, Ph - Phoronida, Ch - Chordata, Ec - Echinodermata, Cn - Cnidaria.

On average, 77% of the assembled sequences were annotated, either with Swiss-Prot matches or by eggnog-mapper (Figure 4). Slightly more sequences received annotations from eggnog-mapper than from Swiss-Prot (63% vs. 59%). The highest mean protein length was only 447 amino acids (AAs), indicating that most translation were likely to be protein fragments (Figure 4). This could be the reason nearly one third of the sequences remain unannotated: these sequences were perhaps too short to generate statistically significant alignments against any of the annotation databases. Another possibility is that Swiss-Prot and EggNOG – being well-curated data sets – do not possess orthologs to many of these unannotated sequences. As such, there may very well be novel and interesting proteins to be discovered in the unannotated data. Unfortunately, in contrast to assembly quality, it is difficult to compare the quality of annotations between studies. For instance, 50% of the sequences from our *Calanus helgolandicus* assembly received annotations from Swiss-Prot. In contrast, the Asai et al. study (95) cited earlier managed to annotate 63.9% of their assembly against Swiss-Prot. But the absolute number of sequences annotated in our study (20,875) is greater than in theirs (19,386). These differences boil down to difference in how the assemblies were filtered, search stringency criteria, and differences in database versions used. For this reason, and given the absence of sequencing data for many species from this study, we are unable to make comparative observations concerning the quality of our annotations.

### Ortholog identification workflow validation

We took advantage of the multiple reference proteomes (fly, butterfly, and mouse) to evaluate how well the OrthoFinder-based approach for identifying candidates based on pairwise orthology worked. Only a single reference sequence from one of the three reference species for each circadian clock component had been used as bait even when all three reference proteomes possessed orthologs for that component. For instance, although all three reference organisms possess a CLK, only *D. melanogaster* CLK was used to identify candidate orthologs. Therefore, additional known circadian clock sequences orthologous to the baits were present in the input data. We examined whether our bait reference sequences were able to acquire these ‘unused reference orthologs’ as pairwise matches; this would serve as an indicator for the sensitivity of the approach.

Encouragingly, the workflow was able to find the correct unused reference ortholog(s) in almost all cases including instances of one-to-many orthology (Table 2). For example, it correctly identified ARNTL and ARNTL2 from mouse as orthologs of fly CYC (37) (Table 2). Importantly, orthologs that are not known to be involved in the circadian clock directly in the host organism were also correctly identified by the corresponding baits. Namely, *D. melanogaster* HR3, which known to be involved in the clocks of some insects (115,116) but not in the fly, was correctly identified as the ortholog of *M. musculus* RORa. Surprisingly, although fly CLK, butterfly CLK, and mouse CLOCK were placed together in in the same phylogenetic orthogroup by OrthoFinder (OG0003270, see supplementary file s1_tree_OG0003270_CLK.pdf), OrthoFinder did not assign mouse CLOCK as an ortholog to either of the insect CLK proteins, making this the only false negative assignment. This hints at a potential sensitivity issue (that may be input data dependent), indicating that orthologs that do exist may be missed. However, as the workflow did not report any false-positive ortholog assignments, we elected to proceed with the rest of the analysis to identify clock protein candidates from our data. A confusion matrix can be found in the supplements (supplementary file s4_orthofinder_confusion_matrix.docx).

To explore this sensitivity issue further, we tested whether the results from OrthoFinder were influenced by the taxonomic diversity of the input data, we re-executed this step with additional proteomes. Namely, we included all metazoan ‘reference’ proteomes available on UniProt (with a BUSCO score of 95% or higher, excluding proteome types ‘Excluded’, ‘Redundant’, ‘Other’; 137 proteomes excluding the three we already had in use) and re-executed OrthoFinder with the exact same parameters including (a now expanded) species tree. We then investigated how many of the candidates sequences from our assemblies identified by the bait sequences were shared between both runs. Our analysis indicated that 177 candidates were shared; this was 75.3% of the union of the two results data sets (supplementary file s5_orthofinder_input_comparisons.docx). 34 candidates (14.5%) were unique to the run with the 137 additional reference proteomes, while 24 candidates (10.2%) were unique to the run without the additional reference proteomes. We hypothesize that these differences in the results is likely the result of differences in the species tree topology (see Section ‘Methods’) leading to pairs of sequences not being resolved as pairwise orthologs in one or the other case.

### Identification of circadian clock proteins

Our analysis revealed the presence of at least one circadian clock component in all 17 assemblies (Table 3; supplementary file s6_cc_cand_sel_pub_table.csv). The sequences of these candidates along together with a table indicating the associated bait ortholog, domains in the candidate sequence, and the best matching Swiss-Prot homolog for the candidate can be found in this publication’s data repository (refer Section ‘Availability of supporting data and materials’). Our workflow was unable to identify candidates for a all clock proteins across the sampled taxa (Table 3). Therefore, as some of the species have publicly available genome and/or transcriptome assemblies (see supplementary file s2_ncbi_assem_counts.csv) we performed sequence searches against these data with our bait proteins as queries using MMseqs2 to identify candidates for these cases. These results are indicated in Table 4, and the candidate sequence accessions can be found in the supplements (supplementary file s7_cc_ncbicomp_results.csv). We have discussed our findings from our workflow and these searches against NCBI data – with species grouped into appropriate phyla for convenience – in the paragraphs below.

**Table 3.** Circadian clock protein candidates identified for the 17 species

		Arthropod								Mammalian	
Phylum	Organism	CLK	CYC	TIM	PER	CRY1	CRY2	PDP1e	VRI	REV-ERBa	RORa
Ar	*Acartia tonsa*	1	1	1	1	1	1	3	1	1	2
Ar	*Acartia clausii*	1	1	1	1	1	1	4	1	1	2
Ar	*Calanus helgolandicus*	1	1	1	2	1	1	3	1	1	1
Ar	*Centropages hamatus*	1	1	2	1	2	1	2	1	1	1
Ar	*Temora longicornis*	1	1	2	1	2	1	3	NA	1	NA
Ar	*Crangon crangon*	1	1	2	NA	NA	1	1	1	1	1
Ar	*Corystes sp*.	1	2	3	4	1	1	1	1	2	3
Ar	*Hyperia sp*.	NA	NA	NA	NA	NA	NA	NA	NA	1	1
Ar	*Podon leuckartii*	2	2	2	2	4	4	2	2	1	2
Ar	*Evadne nordmanni*	1	1	1	1	2	2	NA	1	1	2
An	*Poecilochaetus sp*.	1	1	NA	1	1	1	1	1	1	2
An	*Magelona mirabilis*	1	1	1	1	1	1	1	1	1	1
Ph	*Phoronis muelleri*	NA	NA	NA	NA	NA	NA	1	NA	1	1
Ch	*Oikopleura dioica*	1	1	NA	NA	NA	2	2	NA	NA	NA
Ec	*Asterias rubens*	1	1	1	NA	1	1	1	1	1	NA
Cn	*Rathkea octopunctata*	1	1	NA	NA	NA	1	1	NA	NA	NA
Cn	*Phialella quadrata*	1	1	NA	NA	NA	1	2	NA	NA	NA

Numbers indicate number of candidates found. NAs indicate no candidates found. The bait protein sequence which was used to identify the candidates is indicated as the column header. ‘Arthropod’ and ‘Mammalian’ refer to the type of circadian clock the bait protein sequence originates from (*Drosophila melanogaster* and *Mus musculus* respectively). Circadian clock protein abbreviations as in Table 2. Phylum abbreviations: Ar - Arthropoda, An - Annelida, Ph - Phoronida, Ch - Chordata, Ec - Echonodermata, Cn - Cnidaria

**Table 4.** Circadian clock protein candidates identified via searching available NCBI data

		Arthropod								Mammalian	
Phylum	Organism	CLK	CYC	TIM	PER	CRY1	CRY2	PDP1e	VRI	REV-ERBa	RORa
Ar	*Acartia tonsa*	-	-	-	-	-	-	-	-	-	-
Ar	*Acartia clausii*	-	-	-	-	-	-	-	-	-	-
Ar	*Calanus helgolandicus*	-	-	-	-	-	-	-	-	-	-
Ar	*Centropages hamatus*	-	-	-	-	-	-	-	-	-	-
Ar	*Temora longicornis*	-	-	-	-	-	-	-	NH	-	YES
Ar	*Crangon crangon*	-	-	-	YES	YES	-	-	-	-	-
Ar	*Corystes sp*.	-	-	-	-	-	-	-	-	-	-
Ar	*Hyperia sp*.	ND	ND	ND	ND	ND	ND	ND	ND	-	-
Ar	*Podon leuckartii*	-	-	-	-	-	-	-	-	-	-
Ar	*Evadne nordmanni*	-	-	-	-	-	-	ND	-	-	-
An	*Poecilochaetus sp*.	-	-	ND	-	-	-	-	-	-	-
An	*Magelona mirabilis*	-	-	-	-	-	-	-	-	-	-
Ph	*Phoronis muelleri*	YES	YES	NH	YES	YES	YES	-	NH	-	-
Ch	*Oikopleura dioica*	-	-	NH	YES	YES	-	-	NH	YES	YES
Ec	*Asterias rubens*	-	-	-	YES	-	-	-	-	-	YES
Cn	*Rathkea octopunctata*	-	-	ND	ND	ND	-	-	ND	ND	ND
Cn	*Phialella quadrata*	-	-	ND	ND	ND	-	-	ND	ND	ND

These were proteins for which candidates were not discovered by our OrthoFinder-based workflow. YES - candidate sequence found; NH - candidate not found because no homolog detected in the searched NCBI data; ND - candidate not found because no data was available on NCBI; ‘-’ - candidate discovered already by the OrthoFinder-based workflow. The bait protein sequence which was used to identify the candidates is indicated as the column header. ‘Arthropod’ and ‘Mammalian’ refer to the type of circadian clock the bait protein sequence originates from (*Drosophila melanogaster* and *Mus musculus* respectively). Circadian clock protein abbreviations as in Table 2. Phylum abbreviations: Ar - Arthropoda, An - Annelida, Ph - Phoronida, Ch - Chordata, Ec - Echonodermata, Cn - Cnidaria

#### Arthropods

**Figure 5.**
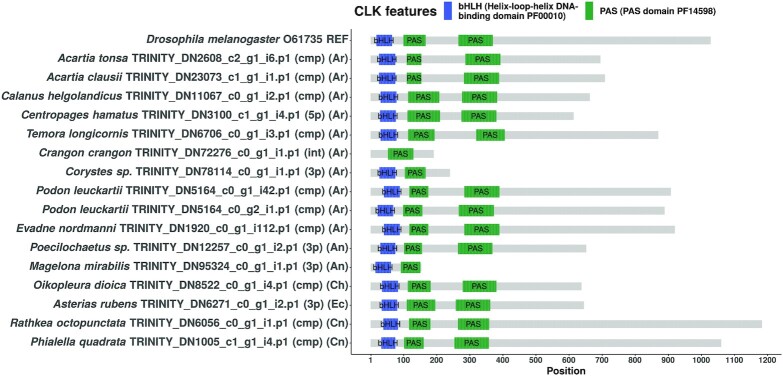
Domain structure diagram of all CLK protein candidates found. The bait sequence used to identify these candidates is the topmost sequence. Annotations in first set of parentheses: cmp = complete sequence, 3p = 3’-partial sequence, 5p = 5’-partial sequence, int = internal fragment sequence. Phylum abbreviations in second set of parentheses: Ar - Arthropoda, An - Annelida, Ph - Phoronida, Ch - Chordata, Ec - Echinodermata, Cn - Cnidaria.

All arthropods in our data set appear to possess canonical or near-canonical circadian clocks. A full complement of orthologs to all core clock components were found in the transcriptomes of *Acartia clausii*, *Acartia tonsa*, *Calanus helgolandicus*, *Centropages hamatus*, *Corystes sp*., and *Podon leuckartii* (Table 3). The branchiopod *Evadne nordmanni*’s candidate set was also nearly complete, with only a candidate for PDP1e being absent. Unfortunately, no assemblies were available on NCBI to validate this absence (Table 4). The calanoid *Temora longicornis* also yielded a full complement of core clock proteins with only a candidate for VRI and RORa missing. However the search against NCBI data indicated that this organism does have a homolog for RORa, indicating that VRI might truly be absent in this organism. The crustacean shrimp *Crangon crangon* was one of the two species for which our workflow did not yield a full set of candidates for the core clock proteins – namely, no orthologs were found for PER and CRY1. However, the search against NCBI data presented suitable candidate sequences for both of these proteins, leaving *C. crangon* with a full complement of clock components. We were unable to find candidate sequences for a majority of the clock proteins in only one arthropod species – the unidentified *Hyperia sp*.. This assembly, which was of exceptionally low quality (Figure 3), yielded orthologs only for REV-ERBa and RORa. Unfortunately, no data was available on NCBI for species from genus *Hyperia* (the species sequenced in our study is unidentified), leaving the portrait of this organism’s clock incomplete. For proteins such as PDP1e and the cryptochromes, some assemblies yielded multiple candidates (Table 3). Interestingly, *P. leuckartii* has a duplicity of candidates for all clock proteins except REV-ERBa. A cursory examination of the two CLK candidates from *P. leuckartii* indicated that both are full length and possess all requisite domains (Figure 5); alignment of the sequences against each other suggested that these are paralogs (see Subsection ‘Duplicity of candidates’ below). Such oddities notwithstanding, the clock setups found here are not only largely identical to those documented in other zooplankton arthropods such as *Daphnia pulex* (74,75), *Daphnia magna* (70,71,76), *Calanus finmarchicus* (77), *Euphausia superba* (78), *Meganyctiphanes norvegica* (79), and *Jasus edwardsii* (80) but also terrestrial arthropods (45,62) as almost all arthropods from our study possess at least one candidate for all clock proteins (especially those that constitute the core TTFL). Therefore, the circadian clock appears to be well-conserved in marine zooplankton arthropods.

#### Annelids and phoronids

Two annelids were sequenced in this study – an unidentified *Poecilochaetus sp*., and *Magelona mirabilis*. Candidate orthologs to all clock components barring PER (absent in *Poecilochaetus sp*.) were found in both species. The only annelid that has a well-characterized circadian clock is *Platynereis dumerilii* (81) which possesses orthologs to all clock components found in arthropods including PER. Unfortunately, we were unable to query NCBI for the missing PER as no data were available at this time for this genus (we sought for assemblies from across the genus as our species is unidentified). It is possible that this unidentified *Poecilochaetus* does carry PER orthologs, and these may surface upon deeper re-sequencing. The absence of PER notwithstanding, our findings effectively triple the number of annelids known to host endogenous circadian clocks, and hint at the conservation of the canonical metazoan circadian clock model in phylum Annelida.

Similar to the annelids in this study the related worm-like phoronid *Phoronis muelleri* also appears to possess a canonical setup. Despite a high quality and highly complete assembly (Figure 1), we were able to find candidate only for REV-ERBa and RORa in *P. muelleri* (Table 3). Unfortunately, very little is known about the circadian clocks of phoronids from literature. Searches against the available NCBI data indicate that candidates for CLK, CYC, PER, CRY1, and CRY2 are likely to be present (Table 4). Homologs for TIM and VRI appears to be absent, suggesting that VRI might not be involved in the circadian pacemaker and the role of TIM being potentially being played by CRY2 (à la some arthropods (45)).

#### Tunicates (chordata) and echinoderms

Our workflow was unable to recover candidates for TIM, PER, and CRY1 from the assembly of the appendicularian tunicate *Oikopleura dioica*. However, sequence searches against available NCBI data suggest that PER and CRY1 do exist, with only TIM being truly absent among the core clock proteins. This is in contrast to the findings from an unpublished manuscript investigating circadian gene expression and ageing which reported that *per* and *cry* genes appear to be entirely absent in the genomes of *Botryllus schlosseri* and 14 other tunicates (133). The same report states that the *cry* gene is absent without distinguishing between insect-type (e.g., fly CRY1) and mammalian-type (e.g., fly CRY2) cryptochromes. However, a comprehensive review on cryptochrome evolution indicates that tunicates do possess CRY2 but not a CRY1 (134) – an observation contested by our findings as the NCBI data indicates that *O. dioica* does have a homolog for CRY1 also. A sequence alignment of this sequence from NCBI against the CRY2 candidates from our workflow suggests that these are entirely different sequences, thereby excluding the possibility of a paralog being mis-assigned (supplementary file s9_oikopleura_dioica_cryptochromes_mafft_aln.fasta). On the other hand, the *O. dioica* clock is also similar to the ascidian tunicate *Ciona intestinalis* in that both species do not appear to have TIM orthologs (135). Similar to *C. intestinalis*, *O. dioica* also appears to possess REV-ERBa and RORa, but not VRI (Table 4). Unlike *C. intestinalis*, which does not seem to possess CLK or CYC (135), our *O. dioica* assembly was able to furnish candidates for both. This suggests that there may be some interesting variations in circadian clocks in the tunicates with the clock of *O. dioica* representing a very canonical variant which only lacks TIM (possibly replaced by CRY2 as in some arthropods (45)) and VRI (potentially not a part of the clock).

We also sequenced the zooplanktonic stage of the sea star *Asterias rubens* (Echinodermata). *A. rubens* appears to have a circadian clock replete with all canonical metazoan components including PER and RORa (which were found by searching against NCBI assemblies). This clock setup appears to be in good agreement with the one discovered in the sea urchin *Strongylocentrotus purpuratus* (136). The only point of contention is the absence of PER in *S. purpuratus* which is present in *A. rubens*. Therefore, *A. rubens* appears to have a canonical circadian clock setup as found in many arthropods.

#### Cnidarians

Both *Rathkea octopunctata* and *Phialella quadrata* possess CLK, CYC, CRY2, and PDP1e. No candidates were found for CRY1 and VRI in either cnidarian; nor were any candidates found for any of the mammalian clock proteins (REV-ERBa and RORa). The cnidarian clock is known to be similar to the arthropod clock, comprising of CLK, CYC, CRY1, CRY2, and VRI (but no PER or TIM) (137). Therefore while the absence of PER and TIM may be unsurprising, CRY1 and VRI not being detected in either assembly is puzzling. Unfortunately no assemblies were available in NCBI databases for these organisms, leaving us to speculate on the circadian clock setup of these species. One explanation could be that the ‘missing’ orthologs for CRY1 and VRI are generally more lowly expressed than the other clock genes in these species, and were left unassembled as a consequence despite the assemblies being highly complete. Alternatively, the two cnidarians might possess very strongly diverged or analogous versions of the missing orthologs, leading to them not being detected by our workflow (which, as indicated earlier, is not 100% sensitive). Although both cnidarians here carry PDP1e, it remains to be seen whether these orthologs are involved in the respective circadian systems. Therefore it appears that *R. octopunctata* and *P. quadrata* may possess clocks typical of cnidarians should candidates for PER and CRY1 be found upon deeper re-sequencing.

### Domain structure visualizations of candidate sequences

We used the seqvisr (124) R package to visualize and compare the domain annotations for the discovered candidates. Domain structure diagrams for CLK, CRY1, and CRY2 are depicted in Figures 5 (CLK), 6 (CRY1), 7 (CRY2) respectively. Visualizations for the remaining clock components can be found in the supplements ( s3_domain_structure_visualizations.pdf). The core functional domains were found to be conserved across all candidates proteins. For instance, all CLK and CYC candidates contain the signature bHLH (basic helix-loop-helix) and PAS (Per-Arnt-Sim) domains at approximately the same locations, with the domains being positioned approximately equidistant from one another across sequences. The same trend is observed in the candidates of the other clock components, although greater variation in inter-domain spacing can be observed for cases such as RORa and TIM. Although some candidates are sequence fragments – in that the transcript was missing the start codon, stop codon, or both – they all possess at least one structured domain expected to be found in that particular clock protein. A manual examination of the functional annotations and ortholog assignments for these candidates revealed no discrepancies (such as unexpected domains) nor false-positive ortholog assignments. In conjunction with the validation of our OrthoFinder-based workflow (Table 2) this suggests that these candidates are very likely to be true orthologs, and therefore functional circadian clock components in the respective organism(s).

**Figure 6.**
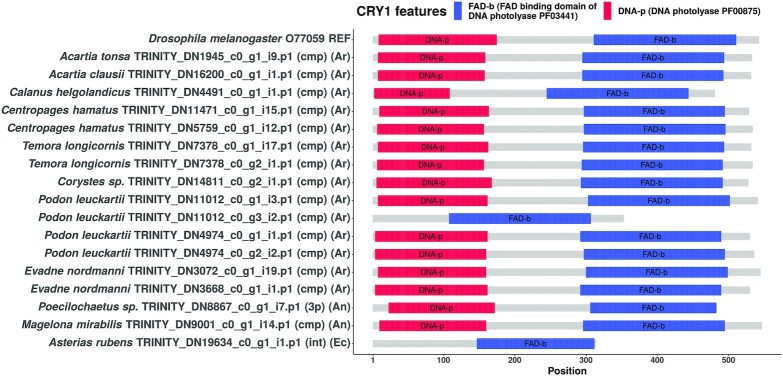
Domain structure diagram of all CRY1 protein candidates found. The bait sequence used to identify these candidates is the topmost sequence. In parentheses: cmp = complete sequence, 3p = 3’-partial sequence, 5p = 5’-partial sequence, int = internal fragment sequence. Phylum abbreviations in second set of parentheses: Ar - Arthropoda, An - Annelida, Ph - Phoronida, Ch - Chordata, Ec - Echinodermata, Cn - Cnidaria.

**Figure 7.**
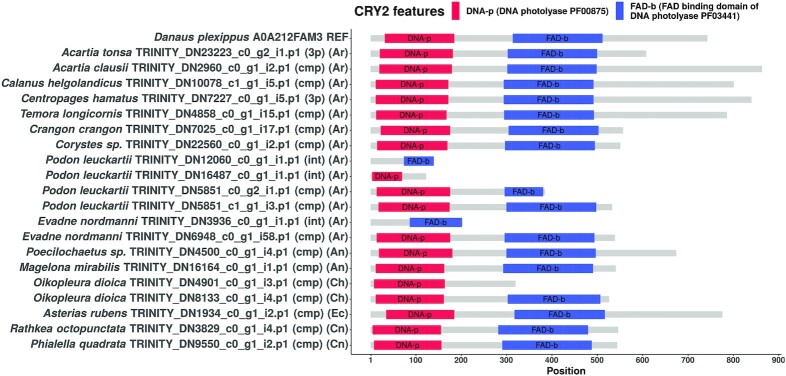
Domain structure diagram of all CRY2 protein candidates found. The bait sequence used to identify these candidates is the topmost sequence. In parentheses: cmp = complete sequence, 3p = 3’-partial sequence, 5p = 5’-partial sequence, int = internal fragment sequence. Phylum abbreviations in second set of parentheses: Ar - Arthropoda, An - Annelida, Ph - Phoronida, Ch - Chordata, Ec - Echinodermata, Cn - Cnidaria.

#### CLK

All CLK candidates – barring two – possess the signature bHLH and PAS domains (Figure 5). The two candidates that did not possess all domains were not full length proteins, i.e., these were translated from ORFs that was lacking the 5ŕegion, 3ŕegion, or both. The bait sequence from *D. melanogaster* is known to be one of the longest CLK proteins, and is only second in length to its counterpart from *Euphausia superba* (> 1300 AA) (78). However, the CLK candidate from *R. octopunctata* is much longer than the *D. melanogaster* CLK. In fact the notion that the *D. melanogaster* CLK is unusual in its length can be dispelled, as multiple CLK candidates from our study are of similar length or even longer (e.g., *P. quadrata* CLK). Additionally, the Q-rich and disordered nature of the C-terminal region found in the *D. melanogaster* and *E. superba* CLK sequences (138)) was observed in many of the candidates (data not shown).

#### CYC

Among the CYC orthologs, the *D. melanogaster* bait appears to be the shortest in length (excluding candidates that are incomplete). All full length candidates possess a bHLH domain followed by two widely spaced PAS domains. *P. leuckartii* appears to have an exceptionally long CYC sequence at almost 900 AA in length. This appears to be the result of an elongated N-terminal region. It is unclear if this elongation is an *in silico* aberration or if it is an actual part of the protein.

#### CRY1 and CRY2

Both cryptochromes are characterized by the presence of a N-terminal DNA photolyase domain and a C-terminal FAD-binding domain (Figures 6 and 7). The cryptochrome candidates were predominantly full length and complete. CRY2 is distinguishable from CRY1 by the presence of a long, disordered C-terminal tail in many (but not all) candidates (data for disorder predictions not provided). This disordered tail has been implicated in the regulation of the circadian clock in mammals (139), and therefore, this role might potentially be conserved in the CRY2 proteins of other metazoans also. While there appears to be much more variation in the length of the CRY2 candidates (mostly from variations in the length of the C-terminal region), the lengths of the CRY1 orthologs is considerably more consistent.

#### PER

Most candidates possess the signature PAS domains and the Per-C terminal domain. Unlike the candidates, the bait PER from *D. melanogaster* itself was not annotated with the Per-C domain by our workflow. Only one candidate was similar to the bait in this regard: a PER candidate from *C. helgolandicus*, Calanus helgolandicus TRINITY_DN15555_c0_g1_i1.p1 (cmp). It is possible that this is an incomplete ORF (covering only the PAS repeat) misidentified by TransDecoder as complete since it is shorter in length in comparison to the other full length candidates and the PER bait. Some candidates were little more than fragments consisting of just a PAS domain or the Per-C domain. Two such candidates were incomplete ORFs from *Corystes sp*. missing both the start and stop codons (Corystes sp TRINITY_DN3394_c0_g2_i1.p1 (int), Corystes sp TRINITY_DN71430_c0_g1_i1.p1 (int)). These can be effectively discarded as the organism possesses another candidate that is full length and complete. The two other PER ‘fragments’ were the sole candidates from *M. mirabilis* and *Poecilochaetus sp*. carrying a PAS domain each respectively (Magelona mirabilis TRINITY_DN57768_c0_g1_i1.p1 (int), Poecilochaetus sp TRINITY_DN40656_c0_g1_i1.p1 (int)). These candidates must be considered with caution as the PAS domain is found in a large variety of proteins (140), and it is possible that these have been misidentified as PER due to their short lengths and incompleteness.

#### TIM

A vast majority of the TIM candidates are full length and complete, and are mostly unremarkable in terms of sequence features. All full length candidates possess possess the TIM N-terminal domain and the C-terminal PAB domain. It can be presumed that all of these candidates also possess the ARM (armadillo) repeats found in canonical TIM proteins (141); these were unfortunately not annotated by our workflow (see Section ‘Methods’). Interestingly, two candidates – one each from *Corystes sp*.(Corystes sp TRINITY_DN12064_c1_g3_i1.p1 (cmp)) and *C. crangon* (Crangon crangon TRINITY_DN7718_c0_g1_i1.p1 (cmp)) – are extremely short (< 300 AA) and comprise solely of the C-terminal PAB domain. It is possible that these are partial sequences despite having been identified as complete by TransDecoder as the tool uses heuristics to identify the coding sequence and is known to be inaccurate (142).

#### PDP1e and VRI

Almost all sequenced species yielded multiple PDP1e candidates, a majority of which were full length proteins. This trend is especially prevalent among the calanoid arthropods (*A. tonsa*, *A. clausii*, *C. helgolandicus*, *C. hamatus*, and *T. longicornis*). All candidates possess the characteristic bZIP domain towards the C-terminal end of the sequence. None of the candidates are as long as the bait sequence from *D. melanogaster*. The candidates for the other bZIP protein, VRI, were mostly unremarkable in comparison, with one exception. Unusually, the VRI candidates from *A. rubens* (Asterias rubens TRINITY_DN754_c0_g1_i2.p1 (cmp)) and *M. mirabilis* (Magelona mirabilis TRINITY_DN8488_c0_g1_i3.p1 (cmp)) carry, not one, but two bZIP domains in contrast to the other candidates (and the bait from *D. melanogaster*) which are all annotated with a single bZIP domain each.

#### REV-ERBa and RORa

Most candidates for the nuclear receptor REV-ERBa possess both the N-terminal Zinc Finger (ZF) domain and downstream Ligand Binding domain (LBD). The full length candidate from *Hyperia sp*.(Hyperia sp TRINITY_DN1096_c0_g1_i4.p1 (cmp)) is unusual as it is missing the ZF domain. Although the candidates do possess the same domains as the bait from *M. musculus* it remains to be seen if they are functional components of the circadian clocks in their respective species. Current evidence indicates that – for instance – in the arthropods the REV-ERBa ortholog(s) mostly function as gas sensors (143), although it is known to be involved in the clock in the firebat *Thermobia domestica* (115). For the other nuclear receptor, RORa, a large number of candidates recovered appear to be internal fragments (i.e., the corresponding ORFs were missing both start and stop codons). Among full length candidates, those from *P. muelleri* (Phoronis muelleri TRINITY_DN2270_c0_g1_i17.p1 (cmp)) and *M. mirabilis* (Magelona mirabilis TRINITY_DN4411_c0_g1_i5.p1 (cmp)) appear to be the most similar to the *M. musculus* bait sequence in terms of length and relative positions of the ZF and LBD domains.

### Duplicity of candidates

Across a number of species, multiple candidates were discovered by our workflow for some clock proteins. For instance the arthropod *Podon leuckartii* has four candidates each for CRY1 and CRY2 (Figures 6 and 7). MSAs of these candidates suggest that these sequences are unlikely to be transcript isoforms not eliminated by our workflow (and/or misclassified by the assembler). They do not appear to be the result of genetic variation either, as the pairwise sequence identities for a vast majority of these is well below 90% (supplementary file s10_ccseqcomp_pid_distribution.pdf). Therefore it appears that these sequences may genuinely be paralogs (at least in the case of the full length sequences). Raw MSAs as well as color-coded visualizations are available in this publication’s data repository (see Section ‘Data and code availability’) under circadian_clock_candidates/multiple_candidate_msa.

## CONCLUSION

Despite being important constituents of marine ecosystems much remains to be discovered about the biology and behavior of zooplankton, and in particular, about their circadian clocks which have been recently implicated in behaviors such as DVM (72) that influence macroscale ecological dynamics. To this end, we sequenced, assembled *de novo*, and annotated the transcriptomes of 17 diverse marine zooplankton. Given the paucity of sequencing data for zooplankton species (82) these high quality annotations and transcriptomes will be valuable resources for the marine ecology community. We then mined these transcriptomes using a phylogenetics-based approach that made use of all the assembled data at our disposal to look for the presence of circadian clock components. We are unaware of any prior work wherein multiple sequences of interest from multiple species were identified simultaneously using *de novo* assembled transcriptomic data in this manner. Additionally, to our knowledge, this is also the first instance of OrthoFinder having been used with transcriptomic data to identify orthlogs of interest. As far as we are aware, it has hitherto only been used to quality-control transcriptomic data (á la Carruthers et al. (144)) or for phylotranscriptomic analysis.

The main limitation of this study is that our findings are based on *in silico*-translated protein sequences derived from *de novo* assembled data sets with less than 25 million reads on average. Therefore, although most assemblies were of high quality based on the quality assessment metrics we used, the absence of a candidate in our results does not imply non-existence of that particular circadian clock component in the host organism as it may have been too lowly expressed to have been sequenced effectively. With some of the sequenced species being meroplanktonic, it is also possible that certain circadian clock mechanisms were inactive or very lowly expressed at this stage, leading to these sequences not being detected by our workflow. It is possible that sequencing transcriptomes from other stages in their lifecycles may provided an extended gene catalog wherein these candidates may be found. Clock gene expression is also driven by the time of the day, and this may have been a contributing factor also as the material may have been collected at a time when the expression levels were low. To rule out the possibility of having missed candidates due to insufficient sequencing in our study, we performed sequence searches against available data on NCBI to identify orthologs for clock components not detected by our workflow in several species (Tables 3 and 4). Unfortunately, despite this effort, we were unable to conclusively establish the composition of the clocks of species such as *R. octopunctata* and *P. quadrata* highlighting the need for further sequencing efforts in these directions. We must note that the candidates discovered via searches against NCBI data were not vetted to the same standard as the candidates in our workflows, and as such may still represent false positives (although this is extremely unlikely). We must also note that our workflow discarded most transcript isoforms in such a way that the trancriptome’s completeness (as measured by BUSCO) remained mostly unaffected while its size and redundancy were significantly reduced; to this end, our workflow selects one ‘representative’ transcript per isoform cluster. However, the retained isoform need not be representative of the biological ground truth as it may contain artefacts incorporated into its sequence that arose during the heuristic assembly process. Therefore, it is possible that the ‘true’, functional circadian clock sequence is among the discarded siblings of a candidate, or is some ‘refined’ version of the selected isoform (e.g., the real transcript is actually shorter at the 3’ end). It is also important to note that our findings are influenced by the limited sensitivity of our ortholog detection workflow (Table 2).

Nonetheless, we managed to identify candidates for most circadian clock components for a majority of the sequenced species. Our findings suggest that the circadian clock composition is conserved across a variety of not only arthropod zooplankton but also annelids, phoronids, chordates, and echinoderms, albeit with potential variations (e.g., TIM being replaced by CRY2). It is only for the two cnidarians – *R. octopunctata* and *P. quadrata* – that we were unable to gather evidence to establish the presence of a canonical metazoan circadian clock. That said, a majority of the candidates found, across species, were full length and replete with the expected functional domains. In cases where a particular component had more than one full length candidate from a species, sequence alignments suggest that these sequences are true paralogs and unlikely to be the result of genetic variation or mis-classification of isoforms. Given that a majority of the species possess candidates for the core components (CLK, CYC, PER, TIM), it is possible that the circadian clocks these proteins would constitute are functionally analogous to their well-characterized counterparts in model organisms. Subsequent efforts in clarifying the status of missing candidates, functional characterization of the discovered sequences *in vivo*/*in vitro*, and identification of the CCG complements will be required to unravel the biological processes under circadian control in these marine zooplankton species.

## DATA AVAILABILITY

The RNA-seq reads and quality controlled final transcriptomes have been deposited at the NCBI under PRJNA824716. The annotation flat files, proteomes, and circadian clock candidate sequences can be found on Zenodo at https://doi.org/10.5281/zenodo.7502741. The source code (BASH and R scripts) along with detailed instructions on how these must be executed can also be found in the aforementioned repository.

## Supplementary Material

lqad007_Supplemental_Files
