# Supplementary material for: RNA sequencing indicates widespread conservation of circadian clocks in marine zooplankton
Source: NAR Genom Bioinform. 2023 Jan 31;5(1):lqad007. doi: 10.1093/nargab/lqad007 (PMC9939569; doi:10.1093/nargab/lqad007)
Supplement: lqad007_Supplemental_Files [file lqad007_supplemental_files.zip › Supplementary Matarial s3_domain_structure_visualizations.pdf]

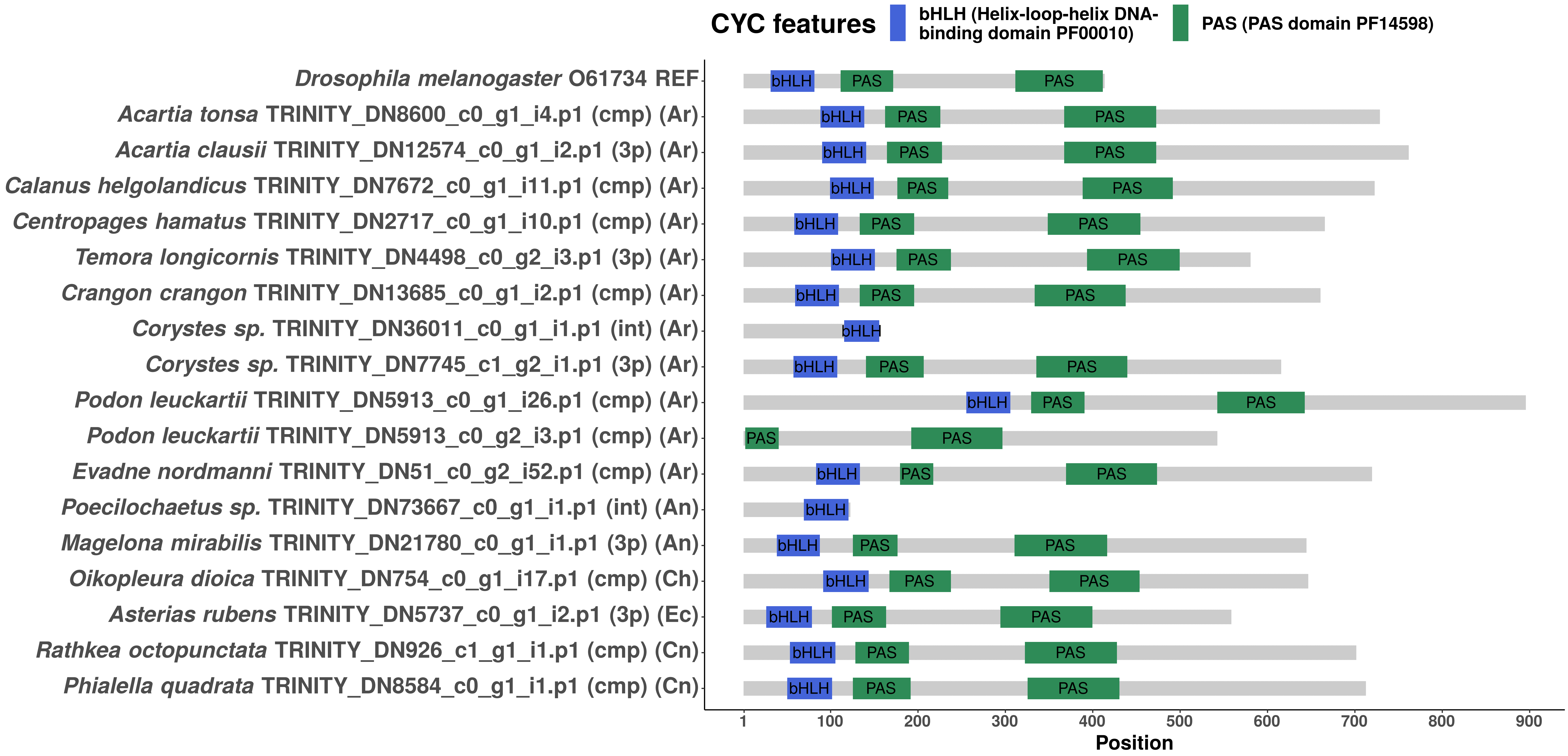
CYC features
bHLH (Helix-loop-helix DNA-binding domain PF00010)
PAS (PAS domain PF14598)
Drosophila melanogaster O61734 REF
Acartia tonsa TRINITY_DN8600_c0_g1_i4.p1 (cmp) (Ar)
Acartia clausii TRINITY_DN12574_c0_g1_i2.p1 (3p) (Ar)
Calanus helgolandicus TRINITY_DN7672_c0_g1_i11.p1 (cmp) (Ar)
Centropages hamatus TRINITY_DN2717_c0_g1_i10.p1 (cmp) (Ar)
Temora longicornis TRINITY_DN4498_c0_g2_i3.p1 (3p) (Ar)
Crangon crangon TRINITY_DN13685_c0_g1_i2.p1 (cmp) (Ar)
Corystes sp. TRINITY_DN36011_c0_g1_i1.p1 (int) (Ar)
Corystes sp. TRINITY_DN7745_c1_g2_i1.p1 (3p) (Ar)
Podon leuckartii TRINITY_DN5913_c0_g1_i26.p1 (cmp) (Ar)
Podon leuckartii TRINITY_DN5913_c0_g2_i3.p1 (cmp) (Ar)
Evadne nordmanni TRINITY_DN51_c0_g2_i52.p1 (cmp) (Ar)
Poecilochaetus sp. TRINITY_DN73667_c0_g1_i1.p1 (int) (An)
Magelona mirabilis TRINITY_DN21780_c0_g1_i1.p1 (3p) (An)
Oikopleura dioica TRINITY_DN754_c0_g1_i17.p1 (cmp) (Ch)
Asterias rubens TRINITY_DN5737_c0_g1_i2.p1 (3p) (Ec)
Rathkea octopunctata TRINITY_DN926_c1_g1_i1.p1 (cmp) (Cn)
Phialella quadrata TRINITY_DN8584_c0_g1_i1.p1 (cmp) (Cn)
bHLH
PAS
1
100
200
300
400
500
600
700
800
900
Position

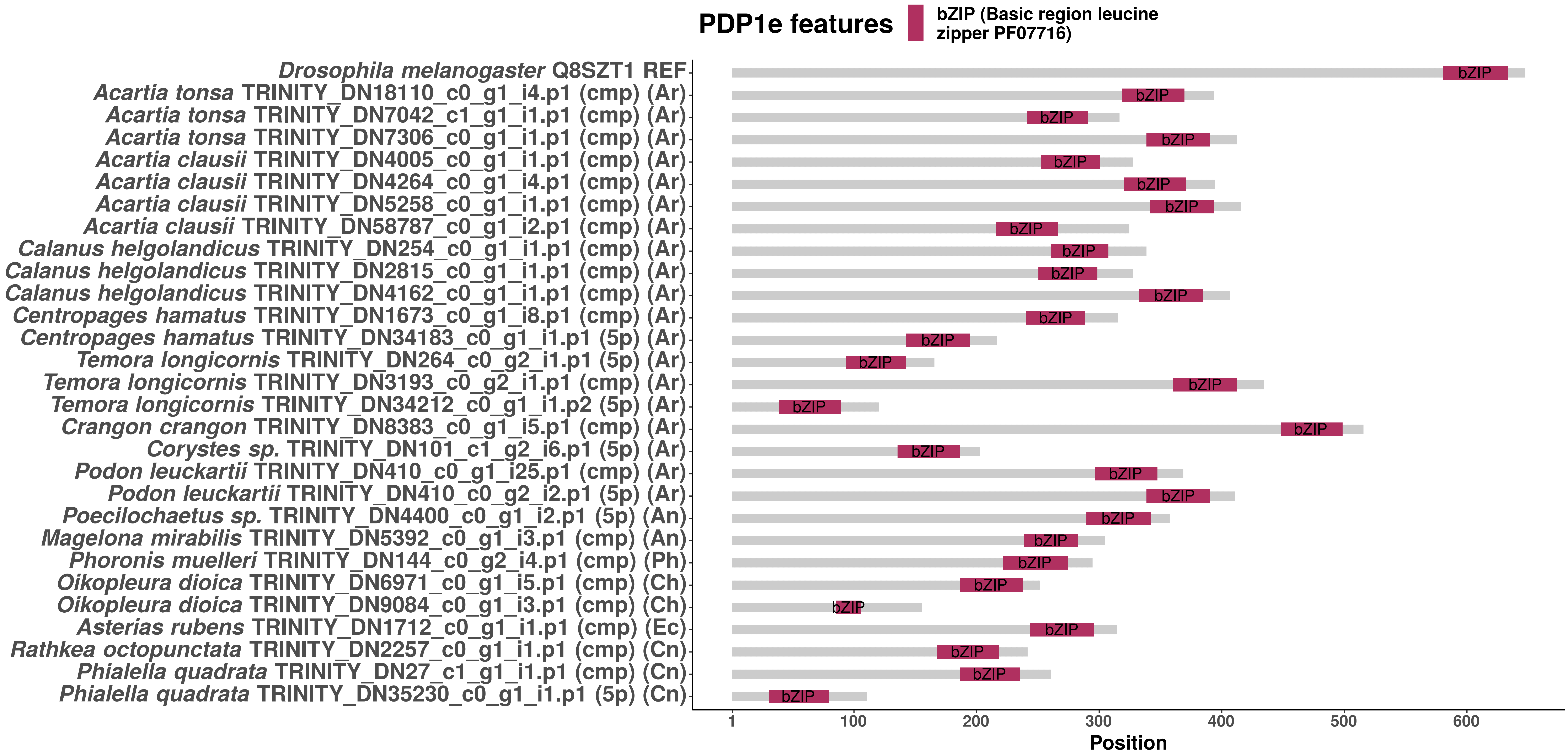
PDP1e features
bZIP (Basic region leucine zipper PF07716)
Drosophila melanogaster Q8SZT1 REF
Acartia tonsa TRINITY_DN18110_c0_g1_i4.p1 (cmp) (Ar)
Acartia tonsa TRINITY_DN7042_c1_g1_i1.p1 (cmp) (Ar)
Acartia tonsa TRINITY_DN7306_c0_g1_i1.p1 (cmp) (Ar)
Acartia clausii TRINITY_DN4005_c0_g1_i1.p1 (cmp) (Ar)
Acartia clausii TRINITY_DN4264_c0_g1_i4.p1 (cmp) (Ar)
Acartia clausii TRINITY_DN5258_c0_g1_i1.p1 (cmp) (Ar)
Acartia clausii TRINITY_DN58787_c0_g1_i2.p1 (cmp) (Ar)
Calanus helgolandicus TRINITY_DN254_c0_g1_i1.p1 (cmp) (Ar)
Calanus helgolandicus TRINITY_DN2815_c0_g1_i1.p1 (cmp) (Ar)
Calanus helgolandicus TRINITY_DN4162_c0_g1_i1.p1 (cmp) (Ar)
Centropages hamatus TRINITY_DN1673_c0_g1_i8.p1 (cmp) (Ar)
Centropages hamatus TRINITY_DN34183_c0_g1_i1.p1 (5p) (Ar)
Temora longicornis TRINITY_DN264_c0_g2_i1.p1 (5p) (Ar)
Temora longicornis TRINITY_DN3193_c0_g2_i1.p1 (cmp) (Ar)
Temora longicornis TRINITY_DN34212_c0_g1_i1.p2 (5p) (Ar)
Crangon crangon TRINITY_DN8383_c0_g1_i5.p1 (cmp) (Ar)
Corystes sp. TRINITY_DN101_c1_g2_i6.p1 (5p) (Ar)
Podon leuckartii TRINITY_DN410_c0_g1_i25.p1 (cmp) (Ar)
Podon leuckartii TRINITY_DN410_c0_g2_i2.p1 (5p) (Ar)
Poecilochaetus sp. TRINITY_DN4400_c0_g1_i2.p1 (5p) (An)
Magelona mirabilis TRINITY_DN5392_c0_g1_i3.p1 (cmp) (An)
Phoronis muelleri TRINITY_DN144_c0_g2_i4.p1 (cmp) (Ph)
Oikopleura dioica TRINITY_DN6971_c0_g1_i5.p1 (cmp) (Ch)
Oikopleura dioica TRINITY_DN9084_c0_g1_i3.p1 (cmp) (Ch)
Asterias rubens TRINITY_DN1712_c0_g1_i1.p1 (cmp) (Ec)
Rathkea octopunctata TRINITY_DN2257_c0_g1_i1.p1 (cmp) (Cn)
Phialella quadrata TRINITY_DN27_c1_g1_i1.p1 (cmp) (Cn)
Phialella quadrata TRINITY_DN35230_c0_g1_i1.p1 (5p) (Cn)
bZIP
1
100
200
300
400
500
600
Position

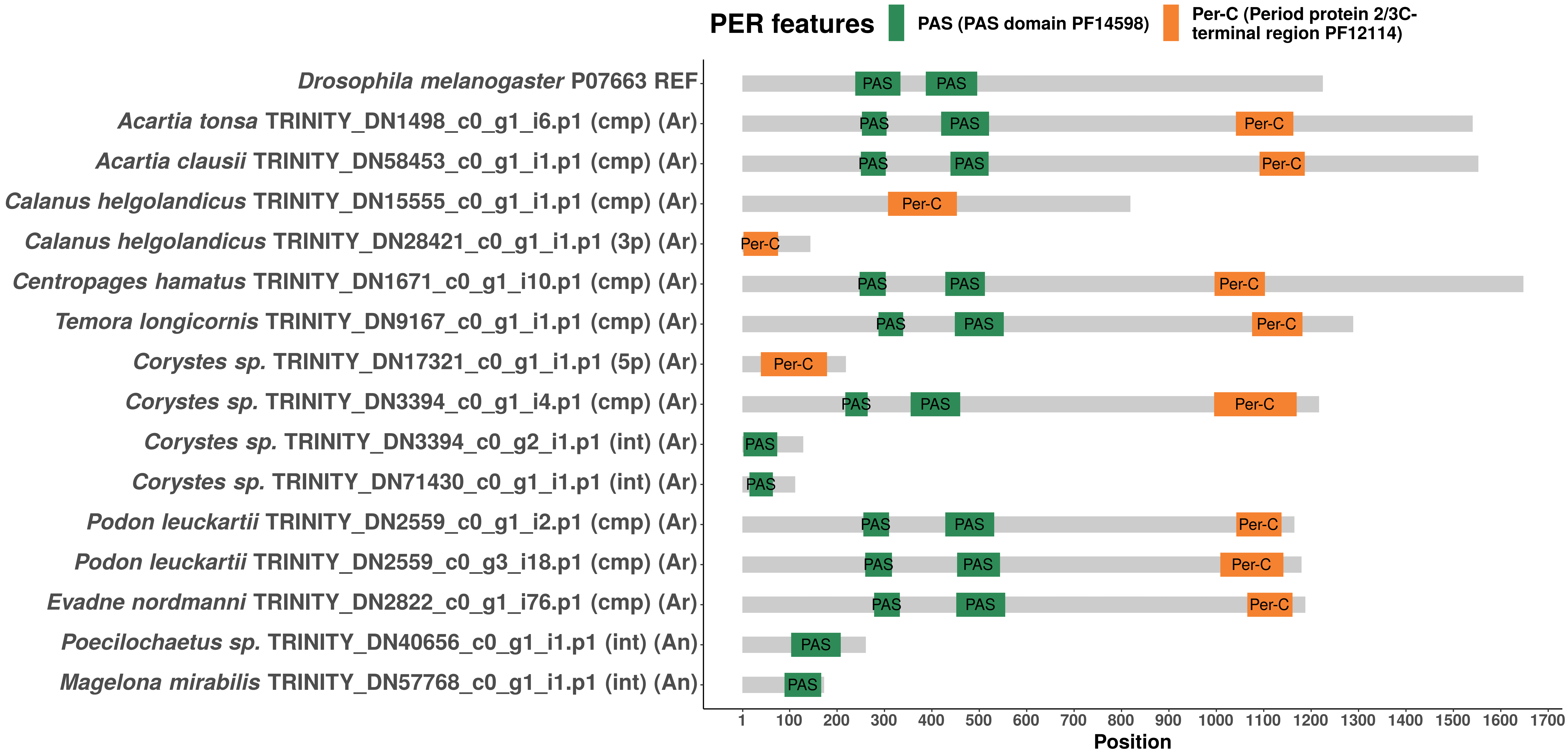

PER features
PAS (PAS domain PF14598)
Per-C (Period protein 2/3C-terminal region PF12114)
Drosophila melanogaster P07663 REF
Acartia tonsa TRINITY_DN1498_c0_g1_i6.p1 (cmp) (Ar)
Acartia clausii TRINITY_DN58453_c0_g1_i1.p1 (cmp) (Ar)
Calanus helgolandicus TRINITY_DN15555_c0_g1_i1.p1 (cmp) (Ar)
Calanus helgolandicus TRINITY_DN28421_c0_g1_i1.p1 (3p) (Ar)
Centropages hamatus TRINITY_DN1671_c0_g1_i10.p1 (cmp) (Ar)
Temora longicornis TRINITY_DN9167_c0_g1_i1.p1 (cmp) (Ar)
Corystes sp. TRINITY_DN17321_c0_g1_i1.p1 (5p) (Ar)
Corystes sp. TRINITY_DN3394_c0_g1_i4.p1 (cmp) (Ar)
Corystes sp. TRINITY_DN3394_c0_g2_i1.p1 (int) (Ar)
Corystes sp. TRINITY_DN71430_c0_g1_i1.p1 (int) (Ar)
Podon leuckartii TRINITY_DN2559_c0_g1_i2.p1 (cmp) (Ar)
Podon leuckartii TRINITY_DN2559_c0_g3_i18.p1 (cmp) (Ar)
Evadne nordmanni TRINITY_DN2822_c0_g1_i76.p1 (cmp) (Ar)
Poecilochaetus sp. TRINITY_DN40656_c0_g1_i1.p1 (int) (An)
Magelona mirabilis TRINITY_DN57768_c0_g1_i1.p1 (int) (An)
PAS
Per-C
1
100
200
300
400
500
600
700
800
900
1000
1100
1200
1300
1400
1500
1600
1700
Position

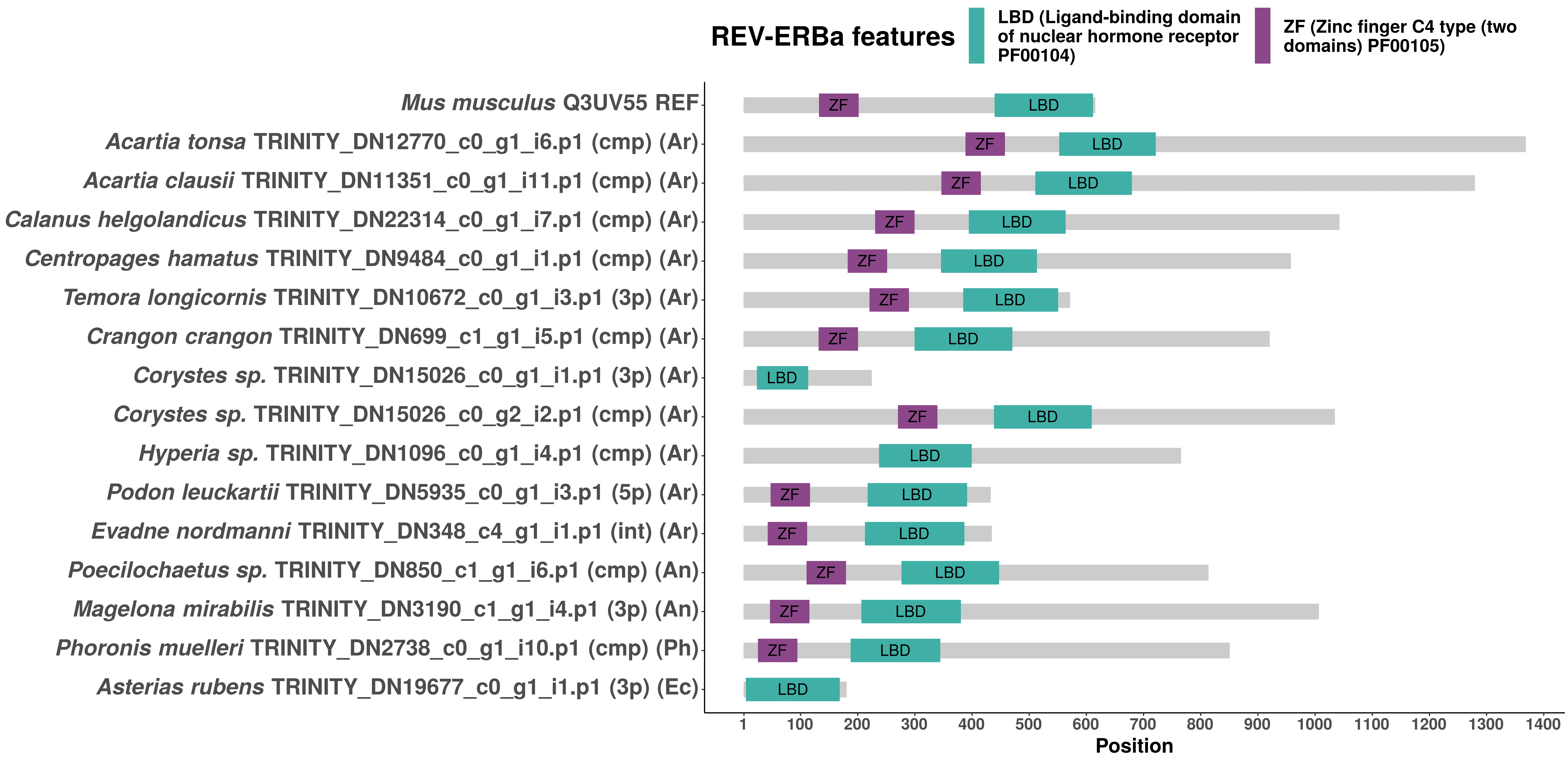
REV-ERBa features
LBD (Ligand-binding domain of nuclear hormone receptor PF00104)
ZF (Zinc finger C4 type (two domains) PF00105)
Mus musculus Q3UV55 REF
Acartia tonsa TRINITY_DN12770_c0_g1_i6.p1 (cmp) (Ar)
Acartia clausii TRINITY_DN11351_c0_g1_i11.p1 (cmp) (Ar)
Calanus helgolandicus TRINITY_DN22314_c0_g1_i7.p1 (cmp) (Ar)
Centropages hamatus TRINITY_DN9484_c0_g1_i1.p1 (cmp) (Ar)
Temora longicornis TRINITY_DN10672_c0_g1_i3.p1 (3p) (Ar)
Crangon crangon TRINITY_DN699_c1_g1_i5.p1 (cmp) (Ar)
Corystes sp. TRINITY_DN15026_c0_g1_i1.p1 (3p) (Ar)
Corystes sp. TRINITY_DN15026_c0_g2_i2.p1 (cmp) (Ar)
Hyperia sp. TRINITY_DN1096_c0_g1_i4.p1 (cmp) (Ar)
Podon leuckartii TRINITY_DN5935_c0_g1_i3.p1 (5p) (Ar)
Evadne nordmanni TRINITY_DN348_c4_g1_i1.p1 (int) (Ar)
Poecilochaetus sp. TRINITY_DN850_c1_g1_i6.p1 (cmp) (An)
Magelona mirabilis TRINITY_DN3190_c1_g1_i4.p1 (3p) (An)
Phoronis muelleri TRINITY_DN2738_c0_g1_i10.p1 (cmp) (Ph)
Asterias rubens TRINITY_DN19677_c0_g1_i1.p1 (3p) (Ec)
ZF
LBD
1
100
200
300
400
500
600
700
800
900
1000
1100
1200
1300
1400
Position

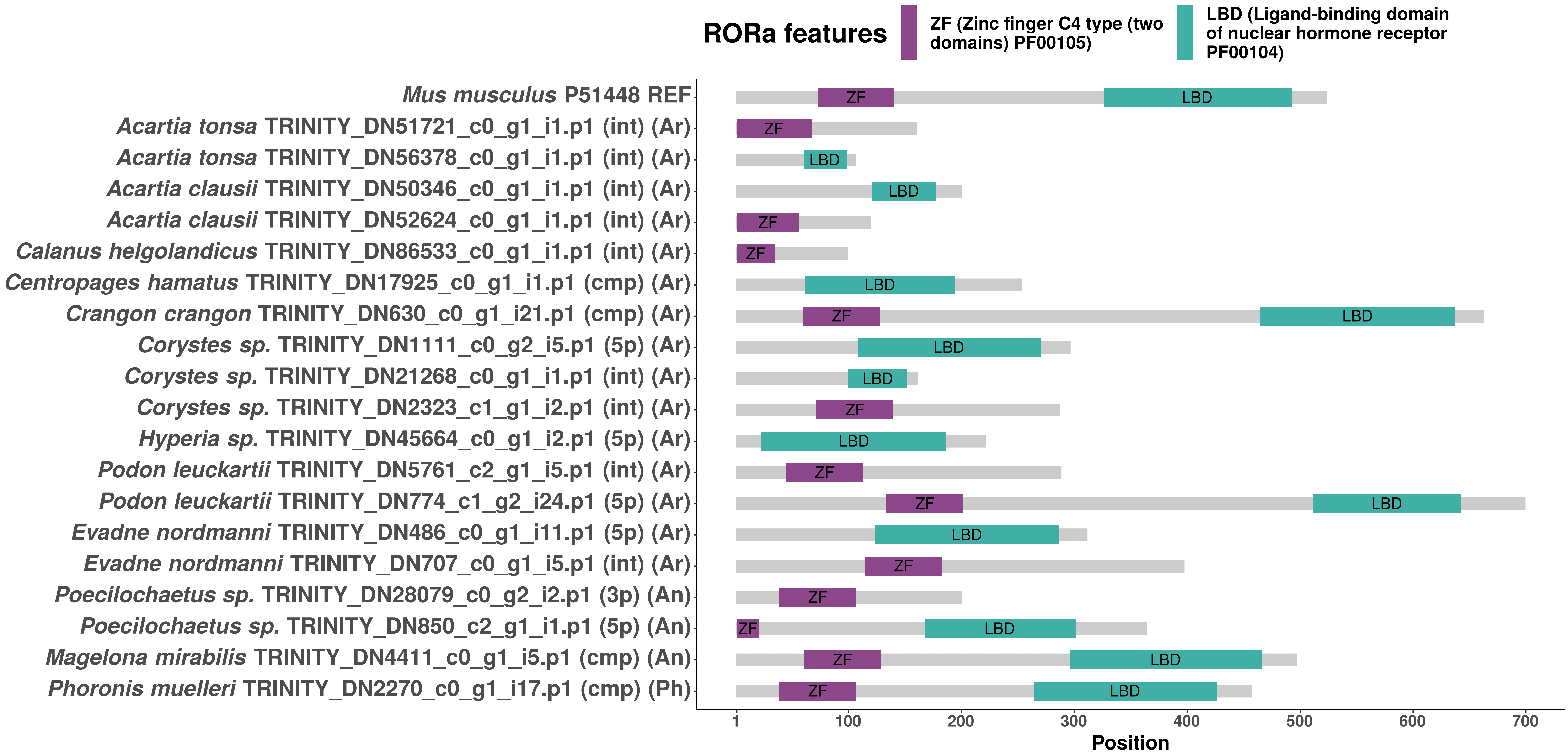
RORa features
ZF (Zinc finger C4 type (two domains) PF00105)
LBD (Ligand-binding domain of nuclear hormone receptor PF00104)
Mus musculus P51448 REF
Acartia tonsa TRINITY_DN51721_c0_g1_i1.p1 (int) (Ar)
Acartia tonsa TRINITY_DN56378_c0_g1_i1.p1 (int) (Ar)
Acartia clausii TRINITY_DN50346_c0_g1_i1.p1 (int) (Ar)
Acartia clausii TRINITY_DN52624_c0_g1_i1.p1 (int) (Ar)
Calanus helgolandicus TRINITY_DN86533_c0_g1_i1.p1 (int) (Ar)
Centropages hamatus TRINITY_DN17925_c0_g1_i1.p1 (cmp) (Ar)
Crangon crangon TRINITY_DN630_c0_g1_i21.p1 (cmp) (Ar)
Corystes sp. TRINITY_DN1111_c0_g2_i5.p1 (5p) (Ar)
Corystes sp. TRINITY_DN21268_c0_g1_i1.p1 (int) (Ar)
Corystes sp. TRINITY_DN2323_c1_g1_i2.p1 (int) (Ar)
Hyperia sp. TRINITY_DN45664_c0_g1_i2.p1 (5p) (Ar)
Podon leuckartii TRINITY_DN5761_c2_g1_i5.p1 (int) (Ar)
Podon leuckartii TRINITY_DN774_c1_g2_i24.p1 (5p) (Ar)
Evadne nordmanni TRINITY_DN486_c0_g1_i11.p1 (5p) (Ar)
Evadne nordmanni TRINITY_DN707_c0_g1_i5.p1 (int) (Ar)
Poecilochaetus sp. TRINITY_DN28079_c0_g2_i2.p1 (3p) (An)
Poecilochaetus sp. TRINITY_DN850_c2_g1_i1.p1 (5p) (An)
Magelona mirabilis TRINITY_DN4411_c0_g1_i5.p1 (cmp) (An)
Phoronis muelleri TRINITY_DN2270_c0_g1_i17.p1 (cmp) (Ph)
1
100
200
300
400
500
600
700
Position

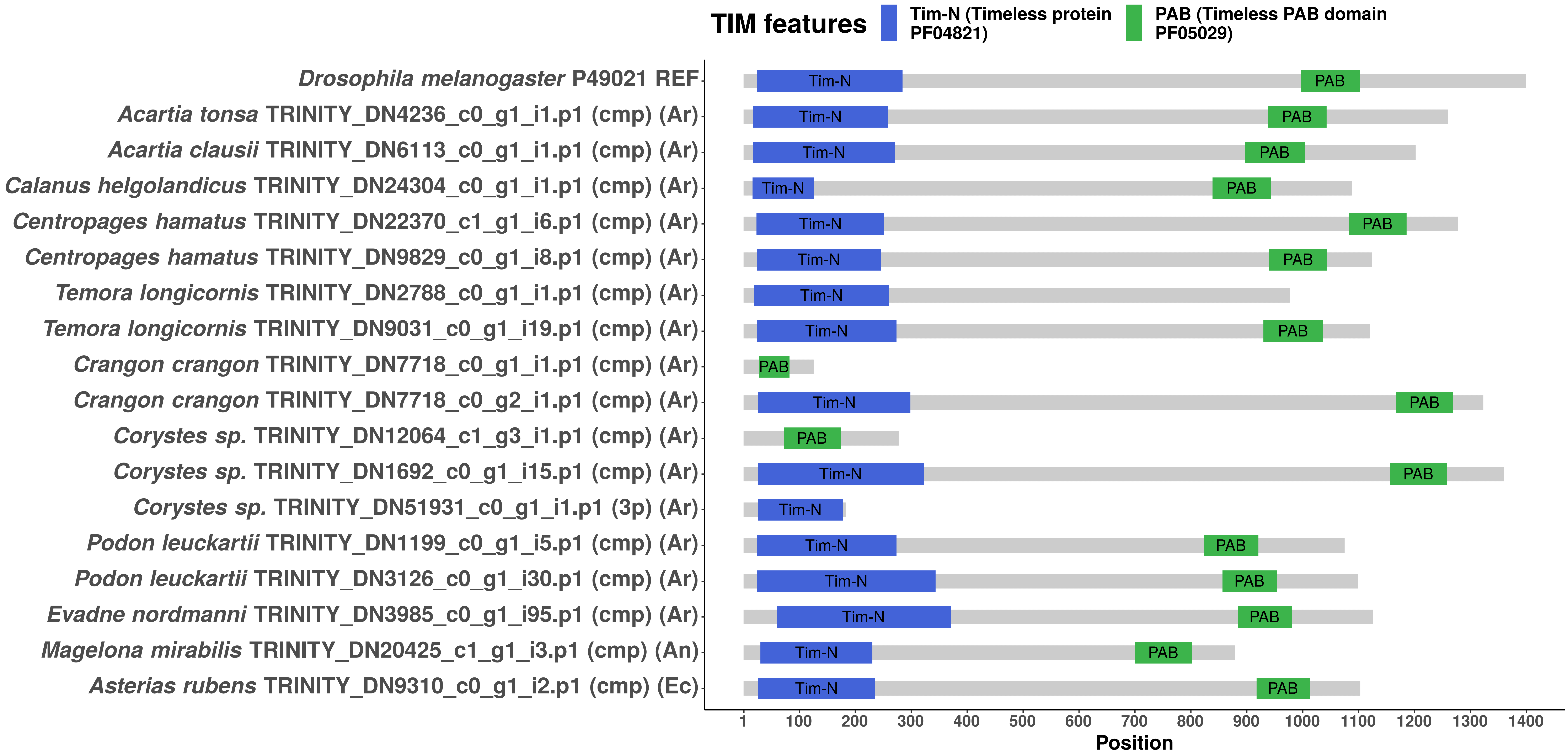

TIM features
Tim-N (Timeless protein PF04821)
PAB (Timeless PAB domain PF05029)
Drosophila melanogaster P49021 REF
Acartia tonsa TRINITY_DN4236_c0_g1_i1.p1 (cmp) (Ar)
Acartia clausii TRINITY_DN6113_c0_g1_i1.p1 (cmp) (Ar)
Calanus helgolandicus TRINITY_DN24304_c0_g1_i1.p1 (cmp) (Ar)
Centropages hamatus TRINITY_DN22370_c1_g1_i6.p1 (cmp) (Ar)
Centropages hamatus TRINITY_DN9829_c0_g1_i8.p1 (cmp) (Ar)
Temora longicornis TRINITY_DN2788_c0_g1_i1.p1 (cmp) (Ar)
Temora longicornis TRINITY_DN9031_c0_g1_i19.p1 (cmp) (Ar)
Crangon crangon TRINITY_DN7718_c0_g1_i1.p1 (cmp) (Ar)
Crangon crangon TRINITY_DN7718_c0_g2_i1.p1 (cmp) (Ar)
Corystes sp. TRINITY_DN12064_c1_g3_i1.p1 (cmp) (Ar)
Corystes sp. TRINITY_DN1692_c0_g1_i15.p1 (cmp) (Ar)
Corystes sp. TRINITY_DN51931_c0_g1_i1.p1 (3p) (Ar)
Podon leuckartii TRINITY_DN1199_c0_g1_i5.p1 (cmp) (Ar)
Podon leuckartii TRINITY_DN3126_c0_g1_i30.p1 (cmp) (Ar)
Evadne nordmanni TRINITY_DN3985_c0_g1_i95.p1 (cmp) (Ar)
Magelona mirabilis TRINITY_DN20425_c1_g1_i3.p1 (cmp) (An)
Asterias rubens TRINITY_DN9310_c0_g1_i2.p1 (cmp) (Ec)
Tim-N
PAB
1
100
200
300
400
500
600
700
800
900
1000
1100
1200
1300
1400
Position

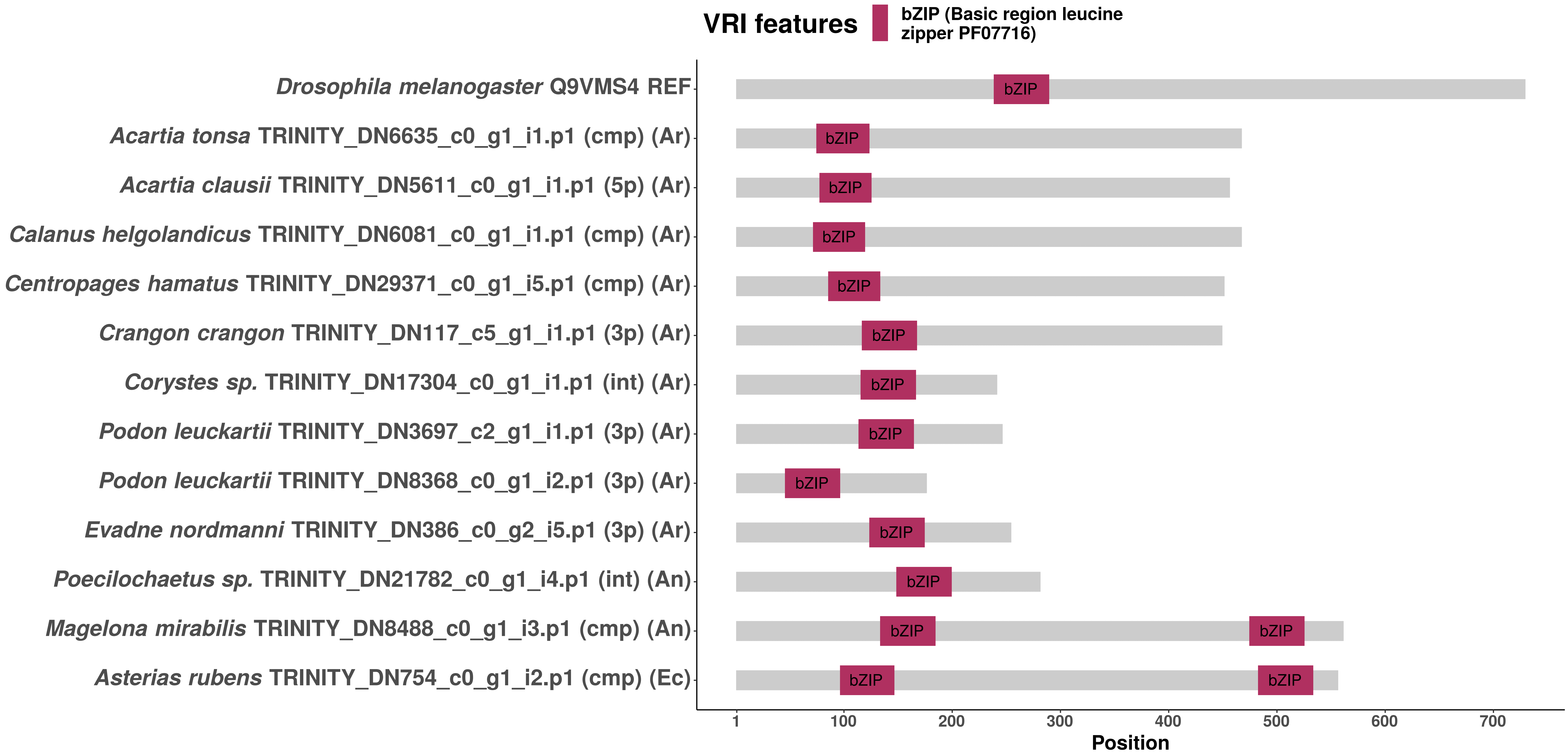
VRI features
bZIP (Basic region leucine zipper PF07716)
Drosophila melanogaster Q9VMS4 REF
Acartia tonsa TRINITY_DN6635_c0_g1_i1.p1 (cmp) (Ar)
Acartia clausii TRINITY_DN5611_c0_g1_i1.p1 (5p) (Ar)
Calanus helgolandicus TRINITY_DN6081_c0_g1_i1.p1 (cmp) (Ar)
Centropages hamatus TRINITY_DN29371_c0_g1_i5.p1 (cmp) (Ar)
Crangon crangon TRINITY_DN117_c5_g1_i1.p1 (3p) (Ar)
Corystes sp. TRINITY_DN17304_c0_g1_i1.p1 (int) (Ar)
Podon leuckartii TRINITY_DN3697_c2_g1_i1.p1 (3p) (Ar)
Podon leuckartii TRINITY_DN8368_c0_g1_i2.p1 (3p) (Ar)
Evadne nordmanni TRINITY_DN386_c0_g2_i5.p1 (3p) (Ar)
Poecilochaetus sp. TRINITY_DN21782_c0_g1_i4.p1 (int) (An)
Magelona mirabilis TRINITY_DN8488_c0_g1_i3.p1 (cmp) (An)
Asterias rubens TRINITY_DN754_c0_g1_i2.p1 (cmp) (Ec)
bZIP
1
100
200
300
400
500
600
700
Position
