# Supplementary material for: RNA sequencing indicates widespread conservation of circadian clocks in marine zooplankton
Source: NAR Genom Bioinform. 2023 Jan 31;5(1):lqad007. doi: 10.1093/nargab/lqad007 (PMC9939569; doi:10.1093/nargab/lqad007)
Supplement: lqad007_Supplemental_Files [file lqad007_supplemental_files.zip › Supplementary Matarial s4_orthofinder_confusion_matrix.docx]

**OrthoFinder confusion matrix based on reference circadian clock proteins only:**

|  | | Ortholog affiliation | |
| --- | --- | --- | --- |
|  |  | True | False |
| Predicted affiliation | True | 18 | 0 |
|  | False | 1 | (452211^a^ - 19) |

^a^ - total number of sequences submitted to OrthoFinder.
