# Supplementary figures and images for: RNA sequencing indicates widespread conservation of circadian clocks in marine zooplankton
Source: NAR Genom Bioinform. 2023 Jan 31;5(1):lqad007. doi: 10.1093/nargab/lqad007 (PMC9939569; doi:10.1093/nargab/lqad007)

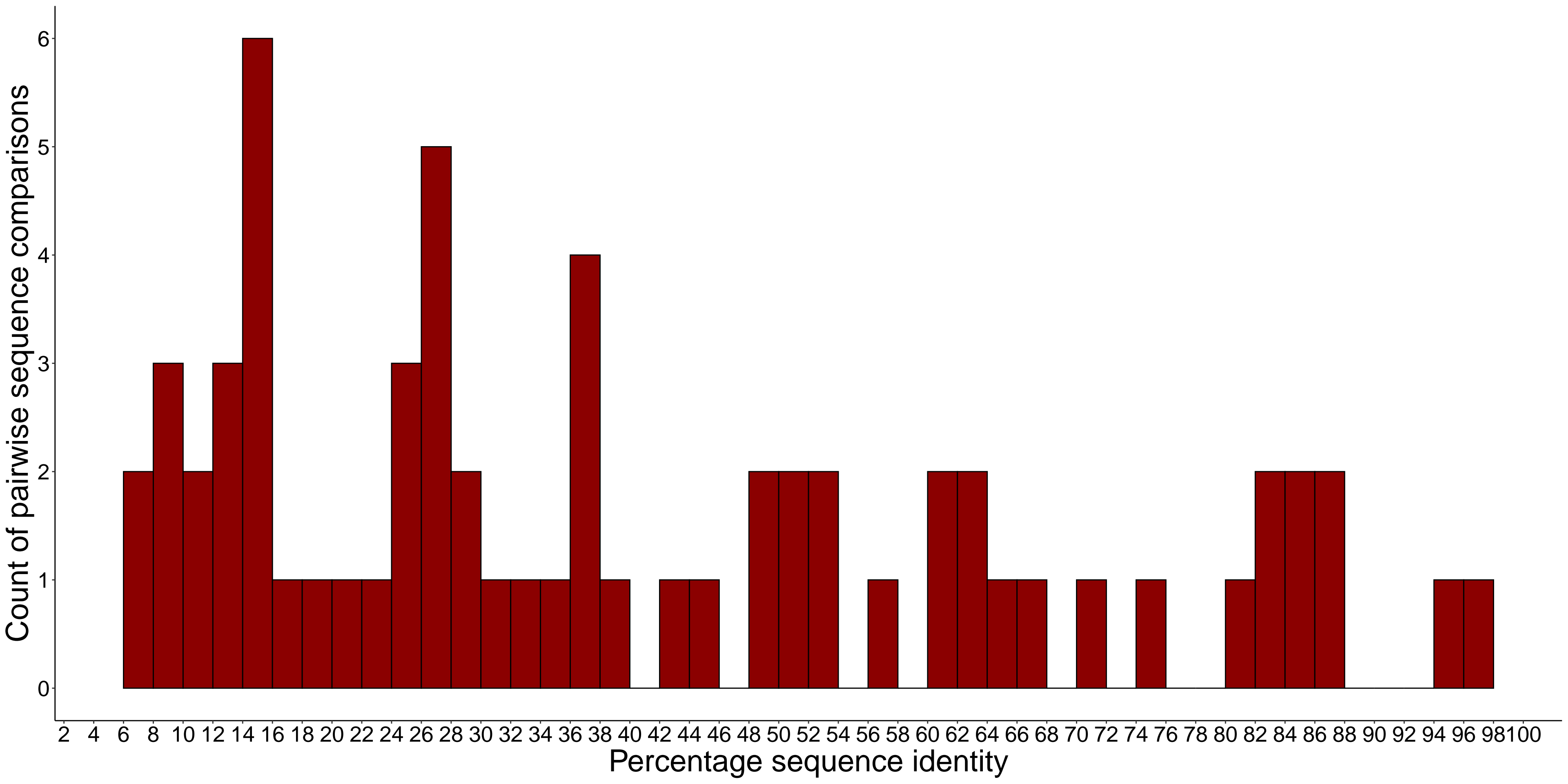

Supplement: lqad007_Supplemental_Files [file lqad007_supplemental_files.zip › Supplementary Matarial s10_ccseqcomp_pid_distribution.pdf]

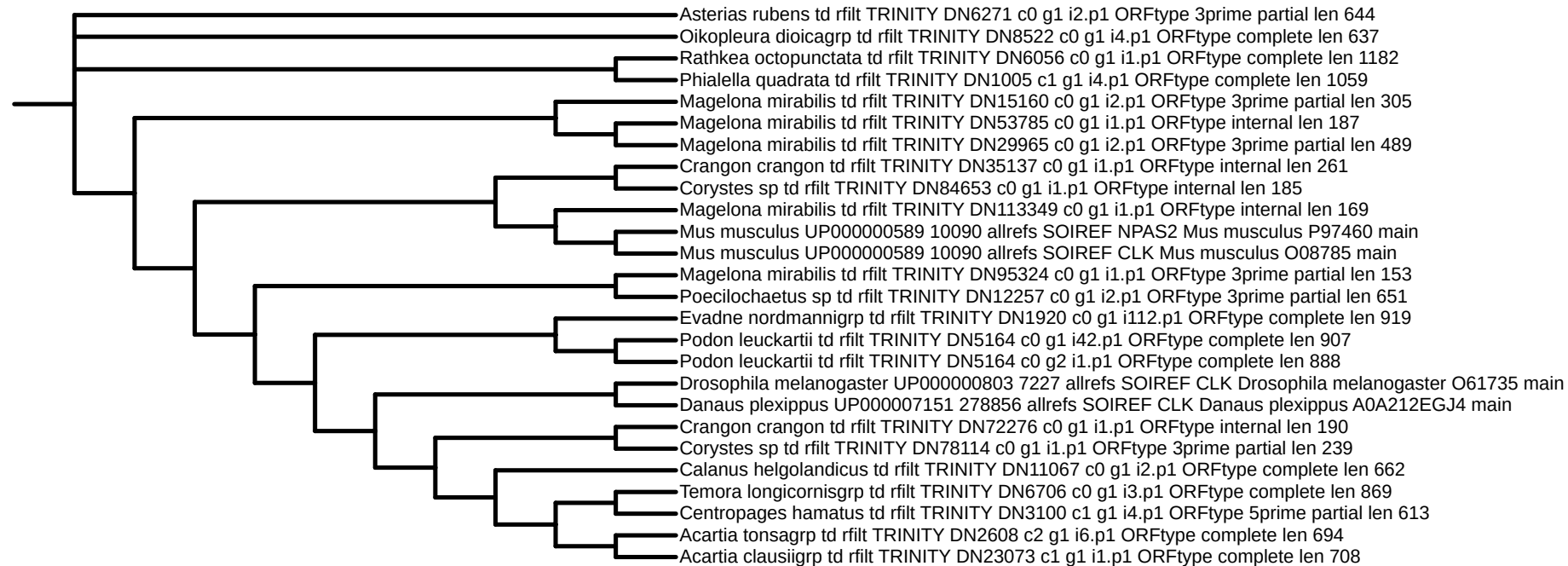

Supplement: lqad007_Supplemental_Files [file lqad007_supplemental_files.zip › Supplementary Matarial s1_tree_OG0003270_CLK.pdf]

With additional reference proteomes (n = 137)

No additional reference proteomes

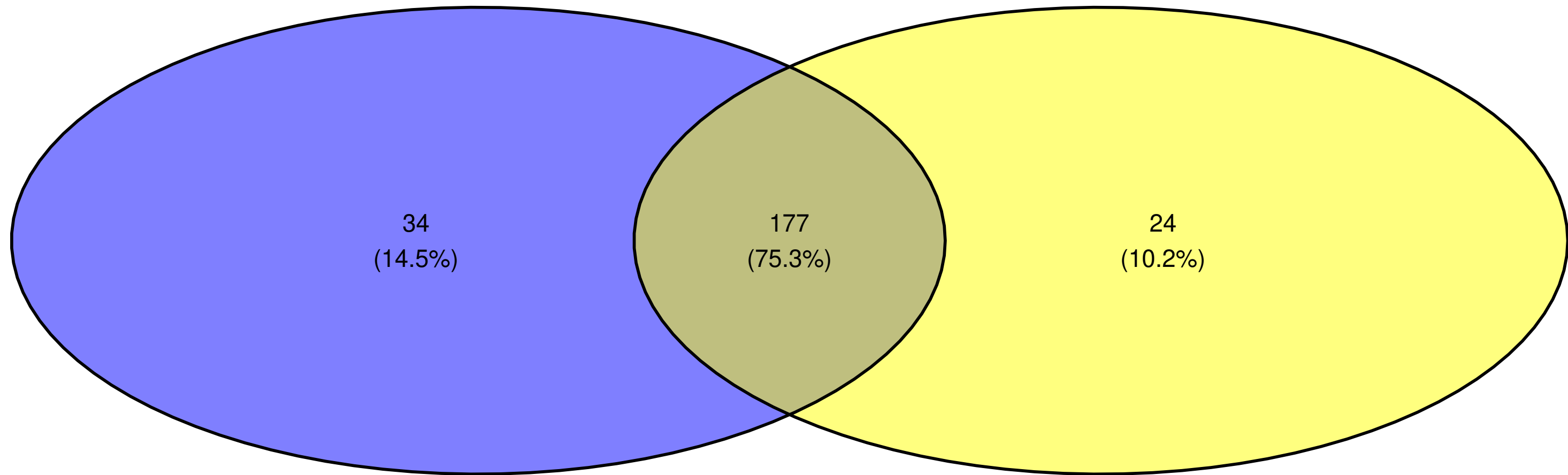

Supplement: lqad007_Supplemental_Files [file lqad007_supplemental_files.zip › Supplementary Matarial s5_orthofinder_input_comparisons.pdf]
